# Core‐Shell Gel Nanofiber Scaffolds Constructed by Microfluidic Spinning toward Wound Repair and Tissue Regeneration

**DOI:** 10.1002/advs.202404433

**Published:** 2024-07-15

**Authors:** Yue Dong, Zongkun Ding, Yuting Bai, Ling‐Yu Lu, Ting Dong, Qing Li, Ji‐Dong Liu, Su Chen

**Affiliations:** ^1^ State Key Laboratory of Materials‐Oriented Chemical Engineering College of Chemical Engineering Jiangsu Key Laboratory of Fine Chemicals and Functional Polymer Materials Nanjing Tech University Nanjing 210009 P. R. China; ^2^ School of Chemical and Environmental Engineering Anhui Polytechnic University Wuhu 241000 P. R. China

**Keywords:** artificial skin, core‐shell gel nanofiber scaffolds, microfluidic spinning, strain sensitivity, wound healing

## Abstract

Growing demand for wound care resulting from the increasing chronic diseases and trauma brings intense pressure to global medical health service system. Artificial skin provides mechanical and microenvironmental support for wound, which is crucial in wound healing and tissue regeneration. However, challenges still remain in the clinical application of artificial skin since the lack of the synergy effect of necessary performance. In this study, a multi‐functional artificial skin is fabricated through microfluidic spinning technology by using core‐shell gel nanofiber scaffolds (NFSs). This strategy can precisely manipulate the microstructure of artificial skin under microscale. The as‐prepared artificial skin demonstrates superior characteristics including surface wettability, breathability, high mechanical strength, strain sensitivity, biocompatibility and biodegradability. Notably, this artificial skin has the capability to deliver medications in a controlled and sustained manner, thereby accelerating the wound healing process. This innovative approach paves the way for the development of a new generation of artificial skin and introduces a novel concept for the structural design of the unique core‐shell gel NFSs.

## Graphical abstract

Multi‐functional core‐shell gel nanofiber scaffolds are prepared by microfluidic spinning technology, showing precisely regulated microstructure, excellent breathability, high mechanical strength, and favorable biocompatibility. Such materials are potentially useful for wound healing and human motion monitoring. This strategy provides a new perspective on the combination of biomedical skin and electronic skin and broadens the application of artificial skin.

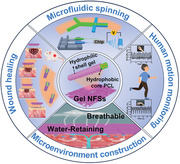

## 1 Introduction

In recent years, microfluidic spinning technology (MST) has garnered increasing attention due to the advancement of fiber spinning chemistry.^[^ ^1^, ^2^ ^]^ This technology holds great promise in the fields of tissue engineering,^[^ ^3^ ^]^ artificial skin^[^ ^4^ ^]^ and biomedicine^[^ ^5^ ^]^ benefitting from the high surface‐to‐volume ratio, superior heat and mass transfer efficiencies and high controllability. Importantly, MST is not only a physical process but also integrates chemical reactions such as chemical crosslinking reaction, photopolymerization reaction and ionic crosslinking reaction.^[^ ^6^, ^7^ ^]^ Furthermore, MST exhibits unique superiorities in precisely regulating the composition and microstructures of materials through using various microfluidic chips, which provides a straightforward platform for the structure design and function integration of advanced materials.^[^ ^8^ ^]^ This strategy plays a vital role in designing and optimizing functional materials, which might promote the iterative upgrading of manufacturing industry.^[^ ^9^, ^10^ ^]^

With the high incidence of chronic diseases such as diabetes and traffic accidents, wound repair has created a huge pressure for global medical health service system. Artificial skin has the potential to promote tissue regeneration and prevent infection and inflammation of wound.^[^ ^11^ ^]^ Nowadays, commercial gel dressings and nanofiber scaffolds (NFSs) dressings have been extensively utilized in clinical due to the similar structure and function to natural skin. Especially for the gel NFSs composites, in which the NFSs provide artificial skin with excellent breathability, high porosity and a large specific surface area,^[^ ^12^ ^]^ and the gel creates a moist microenvironment that supports tissue regeneration.^[^ ^13^, ^14^ ^]^ In this regard, Yuan et al. designed gel NFSs composing of carboxymethyl chitosan‐sodium alginate hydrogel and polymethyl methacrylate nanofiber, which demonstrated great potentials in promoting re‐epithelialization, angiogenesis and tissue regeneration capabilities.^[^ ^15^ ^]^ Lee et al. prepared methacrylate gelatin and polycaprolactone (PCL)/gelatin nanofiber composite, which could regulate the expression of growth factors and collagen, so as to achieve rapid wound contraction and tissue remodeling.^[^ ^16^ ^]^ Zhang et al. designed resveratrol loaded double‐layer gel NFSs composite, which showed great potentials in accelerating hemostasis, hair follicle proliferation and wound healing due to its good compatibility.^[^ ^17^ ^]^ However, conventional approach toward constructing gel NFSs is usually to coat the gel onto NFSs, which results in the inadequate permeability, uncontrollable microstructure and poor mechanical strength of the composites, restricting their practical applications. Therefore, it is on urgent to devise a feasible approach that can effectively integrate the benefits of gel and NFSs, which might hold significant research value and scientific importance for the advancement of artificial skin.

**Scheme 1:**
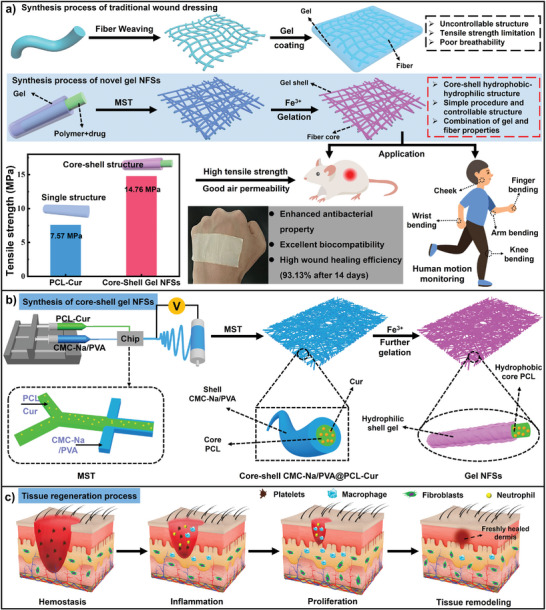
Schematic diagrams of a) the comparison of the conventional and as‐prepared core‐shell gel NFSs, b) fabricating core‐shell gel NFSs through MST and c) tissue regeneration process after treated by the as‐prepared core‐shell gel NFSs.

In this work, we creatively designed a multi‐functional artificial skin through MST by using core‐shell sodium carboxymethyl cellulose‐ferric ion complex/polyvinyl alcohol @ PCL‐curcumin (CMC‐Fe/PVA@PCL‐Cur) gel NFSs (hereinafter referred to as “gel NFSs”) as building blocks. The gel NFSs are composed of hydrophobic PCL‐Cur core and hydrophilic CMC‐Fe/PVA gel shell, maintaining the nanofiber's nanoscale architecture and creates a crucial microenvironment for tissue regeneration by combining reinforced surface wettability and breathability (**Scheme** **1**a). By the means of MST, the microstructure of the core‐shell gel NFSs can be precisely regulated during the spinning process, which lays a foundation for the structure regulation of artificial skin. Based on the rapid chelation of the sodium carboxymethyl cellulose (CMC‐Na) and Fe^3+^, the artificial skin exhibits exceptional mechanical properties and strain sensitivity (Scheme 1b). Additionally, this artificial skin possesses biocompatibility, biodegradability, drug release capabilities and antibacterial properties (Scheme 1c). In vivo experiments on full‐thickness wounds show that this artificial skin achieves a high wound healing efficiency of 93.13% after 14 days of treatment. These features offer the artificial skin with high clinical values in wound healing and tissue regeneration. This work presents a bright prospect for producing new generation artificial skin with superior characteristics, providing a new thought to the development of global medical and healthcare services.

## 2 Results and Discussion

### 2.1 Preparation and Characterization of Gel NFSs

In order to meet the clinical needs of wound healing and tissue regeneration, scientists have been dedicating to realize the functional integration of artificial skin.^[^ ^18^, ^19^ ^]^ For instance, artificial skin is expected to possess surface wettability, porosity, breathability, mechanical strength and biocompatibility, which play crucial roles for facilitating the nutrient transport, promoting the cell adhesion and proliferation and accelerating new blood vessels formation.^[^ ^20^, ^21^ ^]^ Additionally, biodegradability and antibacterial properties are not only essential for preventing bacterial infections, but also can accelerate the healing process.^[^ ^22^ ^]^ These characteristics put forward strict requests for both microstructure regulation and function coupling of artificial skin. To this end, we developed an innovative artificial skin through MST by using novel core‐shell gel NFSs as building blocks, in which the hydrophobic PCL‐Cur and hydrophilic CMC‐Fe/PVA gel are served as core and shell, respectively. MST can precisely manipulate the microstructure of gel NFSs and promote the production efficiency. On the one hand, the multi‐phase fluids flow in a parallel manner in microchannel, preventing adjacent fluids mixing and resulting in a clear demarcation between the core and shell of the nanofibers. On the other hand, the multi‐phase fluid within the microfluidic channel exhibits high mass transfer efficiency, in which the molecular diffusion time can be calculated through the following equation:

(1) $$\tau \;=\frac{L^{2}}{D}\;$$

where *τ*, *L* and *D* represent diffusion time, diffusion length and diffusion constant, respectively. Benefitting from the low diffusion length (the channel width) of microchannel, the molecular diffusion process can be significantly accelerated, which can effectively facilitate the fiber formation.^[^ ^23^, ^24^ ^]^

**Figure 1:**
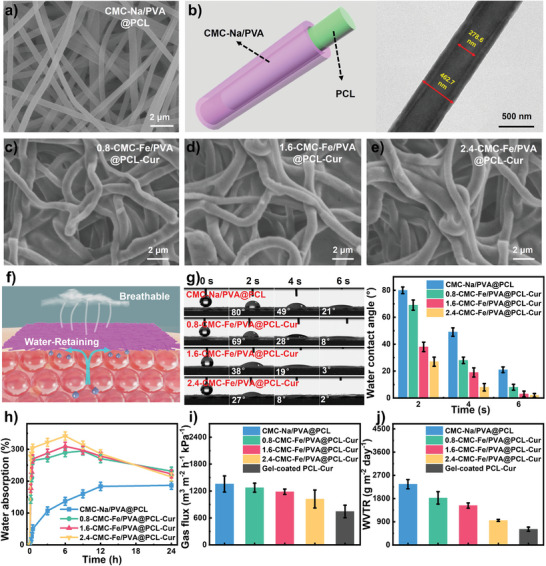
a) SEM image of CMC‐Na/PVA@PCL nanofiber. b) Schematic diagram and TEM image of core‐shell CMC‐Na/PVA@PCL nanofiber. c–e) SEM images of gel NFSs. f) Schematic illustration of the surface wettability and breathability of gel NFSs. g) Water contact angle and h) water absorption ratio of CMC‐Na/PVA@PCL nanofiber and gel NFSs with time. i) Nitrogen permeability and j) WVTR of CMC‐Na/PVA@PCL nanofiber, gel NFSs and gel‐coated PCL‐Cur nanofiber.

The microstructure of core‐shell gel NFSs can be precisely manipulated through adjusting MST parameters. The scanning electron microscope (SEM) image depicted in **Figure** **1**a reveals that the CMC‐Na/PVA@PCL nanofiber displays a smooth surface and uniform distribution of diameter. Transmission electron microscope (TEM) imaging confirms the presence of a distinct core‐shell structure in the CMC‐Na/PVA@PCL nanofiber, in which the diameters of shell and core are 462.7 and 278.6 nm, respectively (Figure 1b). After the gelation between CMC‐Na and Fe^3+^ (Figure 1c–e; Figure S1, Supporting Information), the gel NFSs exhibit an increasing diameter. It is noticed that the concentration of CMC‐Na solution, flow rate of CMC‐Na/PVA mixture solution and the mass ratio between CMC‐Na solution and PVA solution are all significant positively correlated with the diameter of the gel NFSs (Figures S2–S4, Supporting Information). The atomic force microscopy (AFM) images show that the surface roughness of CMC‐Na/PVA@PCL nanofiber decreases after gelation (Figure S5, Supporting Information). These are because the higher CMC‐Fe content generates thicker gel shell, forming more stable and uniform core‐shell microstructure of gel NFSs. These findings provide theoretic basis for precisely regulating the microstructure of core‐shell gel NFSs.

In addition, we tested the chemical structure of the gel NFSs. For the gel shell, the fourier transform infrared (FT‐IR) spectra in Figure S6a (Supporting Information) show characteristic peaks at 1637 and 1486 cm^−1^, which represent the asymmetric stretching vibration and symmetric stretching vibration of ‐COO^−^ in CMC‐Na, respectively.^[^ ^25^ ^]^ After gelation, those two characteristic peaks shift to 1611 and 1456 cm^−1^, respectively. This alteration may be attributed to the disruption of the original carboxyl π bond system due to the chelation of Fe^3+^ and CMC‐Na.^[^ ^26^ ^]^ We also conducted the micro‐IR measurements to further visualize the gelation process (Figure S6b, Supporting Information), where the red regions represent high intensity of functional groups, while the blue regions represent low intensity. It could be observed that the intensity of the ─COO^−^ group on CMC‐Na/PVA@PCL nanofiber is relatively high, while the intensity significant decreases after gelation, suggesting the chelation reaction of Fe^3+^ and CMC‐Na.^[^ ^27^ ^]^ For the PCL‐Cur core, the characteristic peaks at 1732 and 1508 cm^−1^ in the FT‐IR spectra correspond to the stretching vibrations of ─C═O in PCL and C═C in Cur, respectively (Figure S7a, Supporting Information). In addition, the intensities of ─C═O (characteristic absorption peak at 1732 cm^−1^) and ─COO^−^ (characteristic absorption peak at 1294 cm^−1^) were significantly decreased after Cur loading (Figure S7b, Supporting Information), which can be ascribed to the formation of hydrogen bonds between Cur and PCL. Moreover, X‐ray diffraction (XRD) was used to examine the crystallization of PCL, Cur and PCL‐Cur (Figure S8, Supporting Information). Notably, the pure Cur has strong diffraction peaks at 2θ of 8.82°, 14.62°, and 17.08°, indicating the crystallization of Cur.^[^ ^28^ ^]^ Additionally, the pure PCL has diffraction peaks at 2θ in 21.4°and 23.7°, corresponding to the (110) and (200) reflection planes, respectively. The intensities of the diffraction peaks are enhanced after loading Cur, which may be the formation of intermolecular and intramolecular hydrogen bonding interactions that promote the crystallization of PCL nanofibers. In addition, the C 1s X‐ray photoelectron spectroscopy (XPS) spectra of PCL nanofibers before and after loading Cur were shown in Figure S9 (Supporting Information).^[^ ^29^ ^]^ It is found that the C═C peak appears in the XPS spectra of PCL‐Cur nanofibers. These results indicate that Cur has been successfully loaded into PCL nanofibers.

### 2.2 Surface Wettability and Breathability of Gel NFSs

Due to the similar microstructure between the extracellular matrix and gel NFSs, it has garnered significant interest in the field of tissue regeneration.^[^ ^30^ ^]^ For one thing, the large specific surface area and high porosity of NFSs provide numerous cell attachment sites and air transport channels, thereby enhancing cell motility, adhesion, proliferation and differentiation.^[^ ^31^, ^32^, ^33^ ^]^ For another, hydrogels can provide essential microenvironment for tissue regeneration, absorbing wound exudates and maintaining a moist wound environment.^[^ ^34^ ^]^ However, conventional technique that coats the hydrogel onto NFSs might block the pores, cutting off the nutriment and oxygen transport and restricting the tissue regeneration efficiency.^[^ ^35^, ^36^ ^]^ The artificial skin that uses core‐shell gel NFSs as building blocks proposed in this work can overcome this deficiency and satisfy the growing demands in tissue engineering area. This artificial skin combines good surface wettability and exceptional breathability (Figure 1f), which can effectively support tissue regeneration.^[^ ^37^ ^]^ The surface wettability of artificial skin plays a crucial role in cell adhesion, proliferation and differentiation, which is vital for tissue regeneration.^[^ ^38^ ^]^ The gel NFSs demonstrate notable hydrophilic characteristics due to the presence of a CMC‐Fe/PVA gel shell. The water contact angle of the gel NFSs exhibits a significant negative relationship with the CMC‐Fe content (Figure 1g). This trend is primarily attributed to the increasing CMC‐Fe content thicken the gel shell. In addition, the gel NFSs demonstrate rapid water absorption velocity (within 6 s) and exhibits a remarkable water absorption capacity (maximum absorption of 341%) within 6 h (Figure 1h). These results suggest the outstanding water absorption properties of the gel NFSs, which are crucial for artificial skin to effectively absorb exudates to maintain optimal skin moisture level.^[^ ^39^ ^]^ Since the CMC‐Fe/PVA gel was formed surrounding the PCL‐Cur nanofiber instead of filling in the interspace of nanofibers, the gel NFSs exhibit excellent breathability. As shown in Figure 1i, the nitrogen permeability of core‐shell gel NFSs shows a significant decreasing tendency along with the increase of the CMC‐Fe content (1022.4 m^3^ m^−2^ h^−1^ kPa^−1^ when CMC‐Fe content is 2.4 wt.%), yet it's still higher than that of conventional hydrogel coated NFSs (745.2 m^3^ m^−2^ h^−1^ kPa^−1^). Moreover, the water vapor transmittance rate (WVTR) of core‐shell gel NFSs exhibits the same tendency with nitrogen permeability (Figure 1j). With the CMC‐Fe content increases from 0.8 to 2.4 wt.%, the WVTR of the sample gradually decreases from 1825.4 to 952.6 g m^−2^ day^−1^. Even though, the gel NFSs still exhibit a higher WVTR (952.6 g m^−2^ day^−1^ when CMC‐Fe content is 2.4 wt.%) to that of conventional hydrogel coated NFSs (612.1 g m^−2^ day^−1^).

### 2.3 Mechanical Property and Strain Sensitivity of Gel NFSs

**Figure 2:**
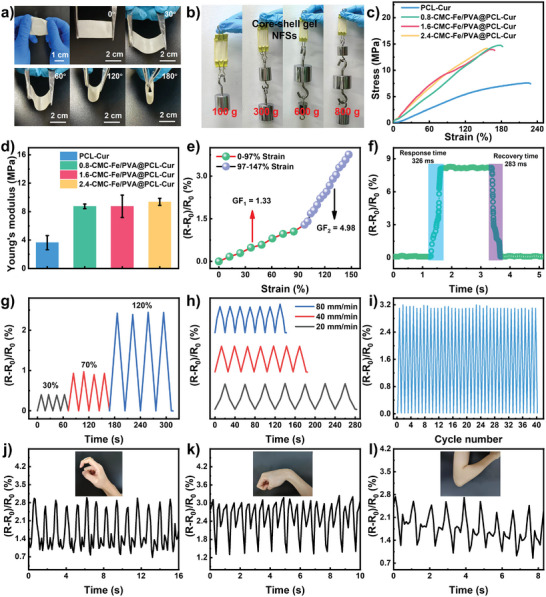
a) Optical images of gel NFSs bent at different angles. b) Optical images of gel NFSs withstanding up to 800 grams of weight without breaking. c) Stress‐strain curves and d) Young's modulus of pure PCL‐Cur nanofiber and gel NFSs. e) Relative resistance changes of the gel NFSs sensor as a function of applied strain. f) Response time and recovery time of the gel NFSs sensor. Relative resistance changes of the gel NFSs sensor under different g) strains and h) stretching velocities. i) Relative resistance changes of the gel NFSs sensor during 40 numbers of cycles bending. Relative resistance changes of gel NFSs sensor with j) finger bending, k) wrist bending and l) arm bending.

Adequate mechanical strength and flexibility are essential requirements for qualified artificial skin, which can ensure that the artificial skin to seamlessly conform to the natural tissue during various movements.^[^ ^40^, ^41^ ^]^ It is noticed that the gel NFSs are flexible enough to bend up to 180° in any direction (**Figure** **2**a) and can withstand at least 800 grams of weight without damage (Figure 2b). Moreover, the gel NFSs exhibit higher tensile stresses than those of the pure PCL‐Cur nanofiber (Figure 2c). Typically, when the CMC‐Fe content is 0.8, 1.6, and 2.4 wt.%, the tensile stresses are 14.7, 13.9, and 14.2 MPa, and the elongations are 177.6%, 160.5%, and 154.5%, respectively (Figure S10, Supporting Information). Furthermore, the Young's modulus of gel NFSs also shows growing tendency along with the increase of CMC‐Fe content (Figure 2d). These might be attribute to the physical crosslinking between Fe^3+^ and ─COO^−^ and the hydrogen bonding interactions within the gel network, which increase the crosslinking density of gel NFSs and improve its mechanical properties.

Apart from the outstanding mechanical behavior, more surprisingly, the gel NFSs also show excellent strain sensitivity. This is attributed to the strain‐induced geometrical effect on the ion transmission.^[^ ^42^, ^43^ ^]^ Specifically, during the deformation of gel NFSs, the length is extended and the cross‐section is shrunk, prolonging the ion transport path, which results in increased resistance.^[^ ^44^, ^45^ ^]^ Benefitting from the outstanding mechanical properties and flexibility, the gel NFSs hold bright prospects for real‐time human motion sensor.^[^ ^46^, ^47^ ^]^ As shown in Figure 2e, the gauge factor (GF) value of the gel NFSs sensor was calculated to be 1.33 over 0 to 97% strain, and rised to 4.98 over 97% to 147% strain, proving the outstanding sensitivity of the gel NFSs sensor.^[^ ^48^ ^]^ And the gel NFSs sensor exhibits 326 ms of response time and 283 ms of recovery time, showing remarkable superiorities in the speed of data transmission (Figure 2f). In addition, the gel NFSs sensor shows steady and reproducible ΔR/R_0_ signals under different deformations (30%, 70%, and 120%) and testing speed (20, 40, and 80 mm min^−1^), demonstrating the excellent stability and reliability in human motion sensor (Figure 2g,h). Moreover, the gel NFSs sensor shows long‐term stability, in which steady ΔR/R_0_ signals are observed over 40 bending cycles (Figure 2i). Given the excellent sensitivity, ultra‐fast response and recovery time, high stability and outstanding reliability, we applied the gel NFSs for real‐time detection of human motion, such as finger bending (Figure 2j), wrist bending (Figure 2k), arm bending (Figure 2l), knee bending and cheek vibration (Figure S11, Supporting Information). The gel NFSs sensor can accurately recognize the motion by exporting different particular ΔR/R_0_ value, in which the ΔR/R_0_ value increases under bending and decreases after release. Therefore, the core‐shell gel NFSs sensor provides a new perspective for the combination of biomedical skin and electronic skin, which might open up a new insight for biomedical skin with sensing function.

### 2.4 Biocompatibility and Biodegradability of Gel NFSs

**Figure 3:**
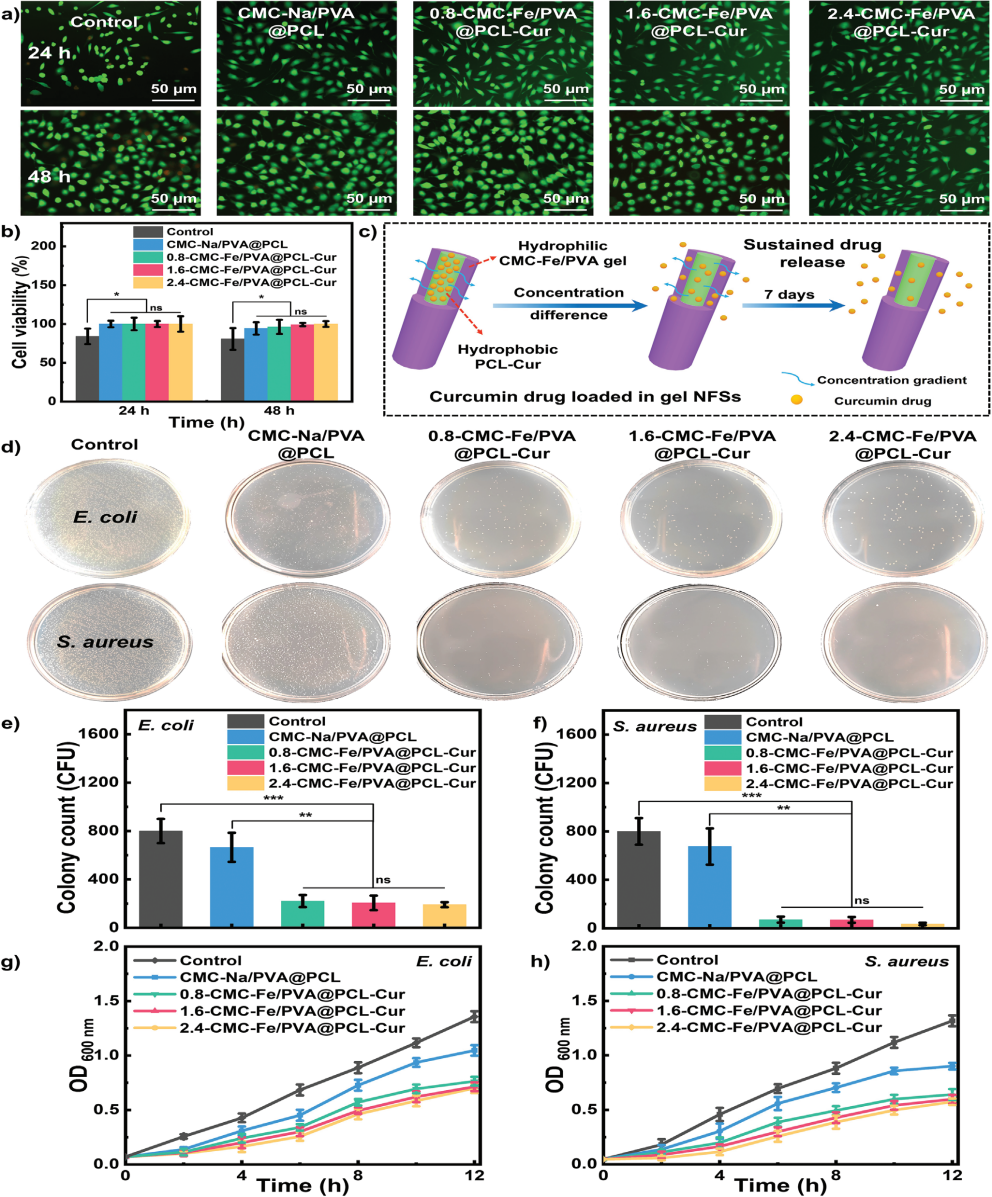
a) Fluorescence micrographs of NIH/3T3 cells proliferating in CMC‐Na/PVA@PCL nanofiber and gel NFSs after 24 and 48 h of culture. b) Cell viability distribution of different samples after 24 and 48 h. c) Mechanism illustration of drug release process. d) Antibacterial property of CMC‐Na/PVA@PCL nanofiber and gel NFSs on against *E. coli* and *S. aureus*. The number of e) *E. coli* and f) *S. aureus* colonies after incubated with different samples. OD_600_ value of g) *E. coli* and h) *S. aureus* suspensions incubated with different samples versus time. Data were shown as mean values ± SD. Bars represent standard error, *n* = 3. *p < 0.05, **p < 0.01, ***p < 0.001.

Biocompatibility is essential for artificial skin to satisfy the requirements of cell to attach, survive and proliferate.^[^ ^49^ ^]^ We inoculated NIH/3T3 fibroblasts onto CMC‐Na/PVA@PCL nanofiber and gel NFSs to assess their biocompatibility. The confocal laser scanning microscopy (CLSM) images in **Figure** **3**a exhibit obvious proliferation of NIH/3T3 cells after incubation on both CMC‐Na/PVA@PCL nanofiber and gel NFSs for 24 and 48 hours, and the cells are uniform spindle shape with clear extended filopodia. Compared with the CMC‐Na/PVA@PCL nanofiber, the gel NFSs show higher cell numbers, and the cell numbers are positively related to the CMC‐Fe content, exhibiting outstanding cytocompatibility of gel NFSs. In addition, Figure 3b reveals that the cell viability of both CMC‐Na/PVA@PCL nanofiber and gel NFSs is higher than that of control group. After culturing for 48 h, cell viability is positively correlated with CMC‐Fe content. The gel NFSs are found to have low cytotoxicity, which can maintain the cell viability level above 90%. Although the gel NFSs can provide temporary support for cellular adhesion, prolonged contact of the gel NFSs with the wound may impede tissue regeneration.^[^ ^50^ ^]^ Therefore, it is crucial for the gel NFSs to undergo degradation in order to facilitate tissue ingrowth.^[^ ^31^ ^]^ Figure S12 (Supporting Information) shows the biodegradability of the gel NFSs. It is observed that the weight of the gel NFSs decreases by 3.84% after 9 days (CMC‐Fe content of 2.4 wt.%), indicating favorable biodegradability of this novel material. Furthermore, the biodegradability of the gel NFSs is enhanced with higher CMC‐Fe content. These results indicate the outstanding biocompatibility and biodegradability, which can provide favorable microenvironment for tissue regeneration.

### 2.5 Drug Release Performance of Gel NFSs

The gel NFSs with drug release capability can significantly speed up the healing process of wounds since Cur has been put into PCL nanofiber. It is worth noting that the release of Cur gradually increases over time, reaching over 70% after 7 days of incubation. Moreover, the Cur release shows negative correlation with CMC‐Fe content (Figures S13 and S14, Supporting Information). These results indicate the sustained drug release behavior of gel NFSs. This might be attributed to the following reasons: 1) The hydrolysis of PCL molecular chain,^[^ ^51^ ^]^ 2) multiple hydrogen bonding interactions between PVA and Cur, 3) the physical crosslinking between CMC‐Na and Fe^3+^. Consequently, the as‐prepared gel NFSs exhibit outstanding sustained drug release performance, providing great potential in wound repair.^[^ ^52^ ^]^ We used zero‐order equation, first‐order equation and Higuchi equation to simulate the kinetics of drug release (Figures S15–S17, Supporting Information).^[^ ^53^ ^]^ The results suggest that the first‐order equation is more consistent with the law of drug release in this research, and the correlation coefficients are all up to 99% (Table S1, Supporting Information). Moreover, the driving force of Cur release is derived from the concentration difference between the nanofibers and the external environment.

We also determined the cumulative release behavior of gel NFSs (CMC‐Fe content of 2.4 wt.%) at different pH conditions (Figure S18, Supporting Information).^[^ ^54^ ^]^ It is noticed that the cumulative release reaches to 30.5%, 24.1%, and 9.2% after 2 days of incubation at pH = 5.0, 6.5, and 7.4, respectively. After 7 days, the maximal Cur release reaches to 75.8% at pH = 5.0. Since the microenvironment of wound infection usually exhibits weak acidity, this new artificial skin is able to achieve cumulative release in case of wound infection. The kinetic equation of the drug release characteristics of the as‐prepared gel NFSs under various pH values was fitted using the Ritger‐Peppas model (Figure S19, Supporting Information).^[^ ^55^ ^]^ It is obvious that the correlation coefficients are all reached up to 95% and the drug release index is 0.72, 0.75, and 0.79 (Table S2, Supporting Information), respectively. According to the drug release index, we can know that this drug release mechanism is mainly a combination of drug diffusion and skeleton dissolution.

### 2.6 Antibacterial Property of Gel NFSs In Vitro

The presence of microbial infections is commonly acknowledged to hinder the wound healing process.^[^ ^56^ ^]^ Therefore, the antimicrobial characteristics of artificial skin have the potential to enhance wound healing by mitigating infections at the site of injury.^[^ ^57^, ^58^ ^]^ In this case, we assessed the antibacterial ability of CMC‐Na/PVA@PCL nanofiber and gel NFSs via using *Escherichia coli* (*E. coli*) and *Staphylococcus aureus* (*S. aureus*). Due to the Cur release (Figure 3c), the gel NFSs show excellent antibacterial properties. Compared with the CMC‐Na/PVA@PCL nanofiber, the number of *E. coli* and *S. aureus* colonies on the gel NFSs decreases significantly after 12 h (Figure 3d). Interestingly, the gel NFSs show more superiority of antibacterial behavior on *S. aureus* than *E. coli*, in which the number of *S. aureus* colonies incubated in CMC‐Na/PVA@PCL nanofiber decreases ≈15.6%, while that incubated in gel NFSs reduces over 90% (Figure 3e,f). And the CMC‐Fe content is negatively correlated with the quantity of *E. Coli* and *S. aureus* colonies. We also measured the OD_600_ value to evaluate the antibacterial activity of the gel NFSs on *E. coli* and *S. aureus* (Figure 3g,h).^[^ ^59^ ^]^ It is noticed that the bacterial suspensions incubated with gel NFSs show lower OD_600_ value compared with that incubated with CMC‐Na/PVA@PCL nanofiber and control group. And the OD_600_ value of bacterial suspensions incubated for 12 h shows negative correlation with the CMC‐Fe content, which is consistent with the tendency of the number of colonies. This excellent antibacterial property might promote the application of the gel NFSs in wound healing.

### 2.7 Applications of Gel NFSs on Promoting Wound Healing and Tissue Regeneration

**Figure 4:**
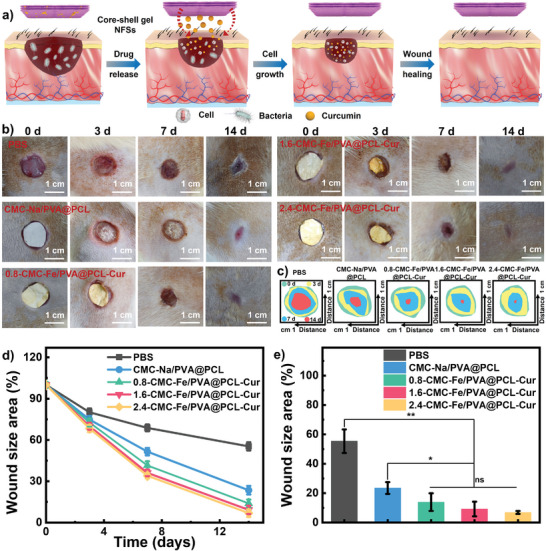
a) Mechanistic diagram of the wound healing process. b) Optical photographs of wounds treated with PBS, CMC‐Na/PVA@PCL nanofiber and gel NFSs. c) Simulated wound shrinkage on 0, 3, 7, and 14 days. d) Wound area changed with time after treated with different samples. e) Wound healing area after 14 days by treated with different samples. Data were shown as mean values ± SD. Bars represent standard error, *n* = 3. *p < 0.05, **p < 0.01.

Base on the synergistic effect of these outstanding characteristics (such as surface wettability, breathability, mechanical strength, strain sensitivity, biocompatibility, biodegradability, drug release and antibacterial capabilities), we have further investigated the practical application of gel NFSs on promoting wound healing and tissue regeneration, aiming at addressing the clinical needs of the of multi‐functional artificial skin.^[^ ^60^, ^61^, ^62^ ^]^ During the wound healing process, the gel NFSs can provide numerous cell attachment sites and nutriment transport channels, creating moist microenvironment, absorbing wound exudates, regulating drug release rate and preventing infection, thereby facilitating wound healing process (**Figure** **4**a). The wound healing efficiency was assessed through measuring the wound area after treated by PBS, CMC‐Na/PVA@PCL nanofiber and gel NFSs with different CMC‐Fe content at intervals of 0, 3, 7, and 14 days. Figure 4b illustrates that the wound treated by gel NFSs with 2.4 wt.% of CMC‐Fe exhibits a notably reduced wound area compared to the other groups, with almost complete healing observed after 14 days. The comparative analysis of wound area reduction following treatment with PBS, CMC‐Na/PVA@PCL nanofiber and gel NFSs is depicted in Figure 4c. Furthermore, when the CMC‐Fe content are 0.8, 1.6, and 2.4 wt.%, the wound areas reduce to 13.91%, 9.22%, and 6.87% after 14 days (Figure 4d). The wound areas observed are notably smaller compared to those treated with PBS (55.36%) and CMC‐Na/PVA@PCL nanofiber (23.49%) as depicted in Figure 4e.

**Figure 5:**
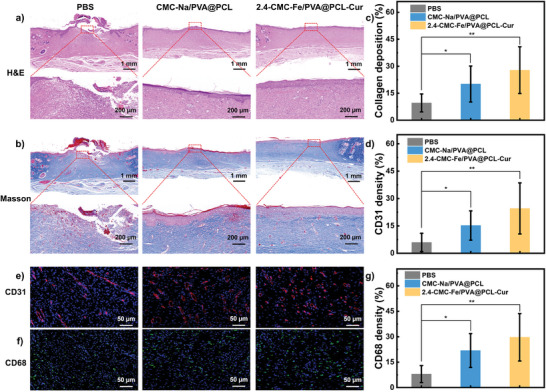
a) H&E staining and b) Masson staining images of the regenerated tissue after treated with PBS, CMC‐Na/PVA@PCL nanofiber and gel NFSs. c) Quantitative statistics of collagen deposition. d) Quantitative statistics of CD31 density. e) CD31 staining and f) CD68 staining images of the regenerated tissue after treated with PBS, CMC‐Na/PVA@PCL nanofiber and gel NFSs. g) Quantitative statistics of CD31 density. Data were shown as mean values ± SD. Bars represent standard error, *n* = 3. *p < 0.05, **p < 0.01.

After that, we performed histological examination through Hematoxylin‐Eosin (H&E) staining and Masson staining.^[^ ^63^, ^64^ ^]^ The regenerated tissue in the control group exhibits irregular and loose structure as evidenced by the H&E staining depicted in **Figure** **5**a. Conversely, the epidermal layers of the regenerated tissue after treated with CMC‐Na/PVA@PCL nanofiber and 2.4‐CMC‐Fe/PVA@PCL‐Cur gel NFSs exhibit a smooth structure with a significantly increased thickness of granulation tissue compared to the control group. Furthermore, the regenerated tissue treated with 2.4‐CMC‐Fe/PVA@PCL‐Cur gel NFSs displays a higher presence of connective tissue and new skin appendages. These findings suggest a significant contribution of 2.4‐CMC‐Fe/PVA@PCL‐Cur gel NFSs in the processes of wound healing and restoration of tissue functionality. The gel NFSs are able to mimic the skin layer during the process of skin tissue regeneration by controlling cell growth and prompting the alignment of collagen fibers. To assess the distribution of collagen fibers on the wound, Masson staining was utilized. Masson staining is a technique that visualizes the distribution of collagen fibers within the wound region. The analysis reveals that the regenerated tissue treated with 2.4‐CMC‐Fe/PVA@PCL‐Cur gel NFSs exhibits a higher density of collagen fibers with intact structure and consistent orientation, as depicted in Figure 5b,c. This observation indicates that the gel NFSs can enhance the generation of collagen fibers. The formation of blood vessels, playing a vital role during the wound healing process, is also characterized by immunohistochemical staining for platelet endothelial cell adhesion molecules (CD31).^[^ ^59^ ^]^ The density of CD31 is notably higher in the 2.4‐CMC‐Fe/PVA@PCL‐Cur gel NFSs group compared to the other groups, indicating that the gel NFSs may promote the formation of new blood vessels (Figure 5d,e). As illustrated in Figure 5f, CD68 serves as a marker for macrophages, playing a pivotal role in the wound healing process by facilitating the removal of bacteria and deceased cells from the wound site.^[^ ^33^ ^]^ Moreover, macrophages contribute to the development of fresh blood vessels, thereby enhancing the wound healing process. Figure 5g demonstrates that macrophages within the 2.4‐CMC‐Fe/PVA@PCL‐Cur gel NFSs group exhibit a higher density, suggesting a remarkable capacity for tissue regeneration. According to these findings, the 2.4‐CMC‐Fe/PVA@PCL‐Cur gel NFSs have enormous potential in tissue regeneration by upregulating the levels of CD31 and CD68, which in turn can speed up the wound healing process.

## 3 Conclusion

In summary, we introduce a straightforward MST method for constructing multi‐functional core‐shell CMC‐Fe/PVA@PCL‐Cur gel NFSs artificial skin. This MST approach can effectively regulate the microstructure of artificial skin. The as‐prepared artificial skin retains the nanofiber's nanostructure and offers a conducive microenvironment for cell attachment, growth and specialization. The unique core‐shell microstructure of gel NFSs endows the artificial skin with superior surface wettability, breathability, mechanical strength and strain responsiveness. Additionally, the artificial skin exhibits remarkable biocompatibility, biodegradability, drug release capabilities and antibacterial properties. These features enable the artificial skin to establish an indispensable microenvironment to promote fibroblast proliferation, leading to a notable 93.13% wound healing efficiency in vivo after a 14‐day treatment period. These promising outcomes affirm that this study not only presents a flexible approach to fabricating core‐shell gel NFSs but also offers fresh perspectives on designing new generation of artificial skin.

## 4 Experimental Section

All materials and methods can be found in Supporting Information.

## Conflict of Interest

The authors declare no conflict of interest.

## Supporting information

Supporting Information

## Data Availability

The data that support the findings of this study are available from the corresponding author upon reasonable request.
