# Supplementary material for: Core‐Shell Gel Nanofiber Scaffolds Constructed by Microfluidic Spinning toward Wound Repair and Tissue Regeneration
Source: Adv Sci (Weinh). 2024 Jul 15;11(39):2404433. doi: 10.1002/advs.202404433 (PMC11497022; doi:10.1002/advs.202404433)
Supplement: Supplementary file 1 — Supporting Information [file ADVS-11-2404433-s001.docx]

Supporting Information

**Core-shell gel nanofiber scaffolds constructed by microfluidic spinning towards wound repair and tissue regeneration**

*Yue Dong^1^, Zongkun Ding^1^, Yuting Bai^1^, Ling-Yu Lu^1^, Ting Dong^1^, Qing Li^1^, Ji-Dong Liu^2^*, and Su Chen^1^**

*Yue Dong^1^, Zongkun Ding^1^, Yuting Bai^1^, Ling-Yu Lu^1^, Ting Dong^1^, Qing Li^1^, Su Chen^1^*

State Key Laboratory of Materials-Oriented Chemical Engineering

College of Chemical Engineering

Jiangsu Key Laboratory of Fine Chemicals and Functional Polymer Materials

Nanjing Tech University

Nanjing 210009, P. R. China

E-mail: chensu@njtech.edu.cn

*Ji-Dong Liu^2^*

School of Chemical and Environmental Engineering

Anhui Polytechnic University

Wuhu 241000, China

E-mail: liujidong8268@ahpu.edu.cn

**Experimental Section**

*Materials:* Polycaprolactone (PCL), polyvinyl alcohol 210 (PVA 210), iron nitrate, curcumin (Cur) and PBS buffer were purchased from Aladdin Reagent Co. (China). Dichloromethane (DCM), acetic acid (HAC), dimethyl sulfoxide (DMSO) and sodium carboxymethyl cellulose (CMC-Na) were obtained from Shanghai Macklin Regent Co., Ltd. (China). Ultrapure water (18 MΩ cm) was used throughout the experiments. All reagents were used directly without additional treatments.

*Microfluidic spinning fabricating core-shell gel NFSs:* The core-shell gel NFSs were constructed through using a coaxial microfluidic electrospinning chip. Typically, the external phase solution (shell material) was obtained through mixing the 12 wt% of PVA aqueous solution and certain concentration of CMC-Na aqueous solution (0.8, 1.6 and 2.4 wt%) at mass ratio of 4 : 1. The internal phase solution (core material) was prepared by dissolving PCL and Cur into the mixed solvent of DCM, HAC and DMSO (DCM : HAC : DMSO = 7 : 2 : 1 v/v/v), in which the mass ratio of PCL and Cur were 10 wt% and 5 wt%, respectively. The as-prepared solutions were injected into the coaxial microfluidic chip at a flow rate of 2 : 1 in the ratio of external phase to internal phase. In addition, the electrospinning voltage and the distance between the coaxial microfluidic chip were 17 kV and 10 cm, respectively. The whole procedure of coaxial microfluidic electrospinning was carried out for 5 h. The as-prepared CMC-Na/PVA@PCL-Cur nanofiber was then immersed in 2 wt% of Fe(NO_3_)_3_ aqueous solution for 5 minutes, allowing the physical crosslinking of CMC-Na, obtaining of CMC-Fe/PVA@PCL-Cur gel NFSs. The excess Fe^3+^ was removed through equilibrating the gel NFSs in deionized water. Finally, the prepared gel NFSs were vacuum dried for 2 days in a constant temperature chamber to obtain the coaxial gel NFSs. The samples constructed by different concentration of CMC-Fe were denoted as 0.8-CMC-Fe/PVA@PCL-Cur, 1.6-CMC-Fe/PVA@PCL-Cur and 2.4-CMC-Fe/PVA@PCL-Cur according to the CMC-Fe content.

*Water absorption performance test:* The dried CMC-Fe/PVA@PCL-Cur gel NFSs (20×20 mm^2^) was soaked into 10 mL PBS buffer solution and incubated at 37 °C. At defined time intervals, the samples were taken out and weighted after wiping the excess PBS solution. Water absorption was calculated based on equation

| $Water absorption \left( \% \right)=\frac{W_{1}-W_{0}}{W_{0}}\times100\%$ | (1) |
| --- | --- |

where *W_1_* and *W_0_* are the weights of the absorbed and dried samples, respectively.

*Nitrogen permeability rate test:* The breathability of core-shell gel NFSs was tested based on previously reported literature. The gas permeability calculation formula was as follows:

| $\Psi=\frac{\eta Qt}{A\Delta P}$ | (2) |
| --- | --- |

where *Ψ* denotes gas permeability (m^2^), *Q* denotes gas flow rate (m^3^ s^-1^), *η* denotes nitrogen viscosity (the viscosity at 25 ℃ is 1.822×10^-5^ Pa s), *t* denotes sample thickness (m), *A* denotes test area (m^2^), and *ΔP* denotes pressure difference between the two sides of the sample (Pa).

*Water vapor transmission rate (WVTR) test:* The WVTR was calculated according to ASTM E96 testing standard. Briefly, the samples were covered onto a round cylindrical cup containing distilled water. And the entire system was placed in a 37 ℃ of temperature and 50% of relative humidity condition. The WVTR was calculated according to the equation

| $WVTR (g/m^{2}/day)=\frac{G}{t\times A}\times24$ | (3) |
| --- | --- |

where *G* (g) represents the mass change of the cylindrical cup in *t* (h) test time and *A* means the effective test area of the cup mouth (m^2^). Each sample was tested 3 times and the average value was taken as the WVTR of the sample.

*Sensing performance characterization:* Resistance was tested using digital multimeter. The change of the electrical signal of the human body under different motion states was detected by the CGS-8 Intelligent Sensing Analysis System. The relative resistance change was calculated by the following formula: ΔR/R_0_ = (R-R_0_)/R_0_, where R_0_ is the initial resistance and R is the change resistance. Gauge factor (GF) was calculated by the following formula: GF = (ΔR/R_0_)/ε = [(R-R_0_)/R_0_]/ε, where ε refers to the applied strain.

*In vitro cell culture:* NIH/3T3 fibroblasts were used in investigate the biocompatibility and cytotoxicity of the gel NFSs. First, the CMC-Fe/PVA@PCL-Cur gel NFSs were pre-treated by ultraviolet light for 30 min and placed in 24-well plates. Afterwards, the cells were seeded onto the gel NFSs with a density of 3×10^4^ cells per well and cultured (37 ℃, 5% CO_2_ humid atmosphere) in Dulbecco’s Modified Eagle Medium containing 10% fetal bovine serum and 1% penicillin-streptomycin. The viability of the cells was studied by using the live-death method. The cells were stained with calcified AM and propidium iodide. The survival of the cells in the medium was observed under a fluorescent microscope, in which the green and red fluorescence represented the live and dead cells, respectively.

*In vitro degradation test:* The pre-weighted CMC-Fe/PVA@PCL-Cur gel NFSs were immersed into PBS buffer solution in a 37 °C shaking bath. At regular intervals, the samples were rinsed with deionized water three times and freeze-dried for weighting. Finally, the degradation behavior was evaluated according to the curves of the weight loss ratio with respect to time.

*In Vitro Drug Release Study:* In order to investigate the drug release performance of CMC-Fe/PVA@PCL-Cur gel NFSs, we first determined the standard curve of Cur concentration (12.5, 10, 5, 2.5, 1.25 and 0.78125 μg mL^-1^) versus the corresponding UV absorbance at 425 nm wavelengths. After that, 90 mg of as-prepared CMC-Fe/PVA@PCL-Cur gel NFSs were immersed into PBS buffer (50 mL) and incubated at 37 °C. 3 mL of release medium was drawn out and 3 mL of fresh PBS was added back for continuing incubation at specific time. The UV absorbance of the release medium was measured at 435 nm wavelengths by using a double-beam UV-Vis spectrophotometer (00135001, model TU-1900). Finally, the cumulative release of Cur was determined according to the standard curve.

*In vitro antimicrobial test: Escherichia coli* (*E. coli*) and *Staphylococcus aureus* (*S. aureus*) were used to investigate the antimicrobial activity of gel NSF. First, *E. coli* and *S. aureus* were incubated in Lysogeny broth (LB) liquid medium (5 mL) at 37 °C. After 24 h of incubating, *E. coli* and *S. aureus* suspension were diluted to the concentration of 10^8^ colony-forming unit (CFU) per mL with saline, respectively. To further investigate the antibacterial properties of the gel NFSs, CMC-Na/PVA@PCL nanofiber, 0.8-CMC-Fe/PVA@PCL-Cur gel NFSs, 1.6-CMC-Fe/PVA@PCL-Cur gel NFSs and 2.4-CMC-Fe/PVA@PCL-Cur gel NFSs were sterilized under ultraviolet light for 30 min. After that, 20 mg of gel NFSs with different CMC-Fe contents and PBS solution were added into 10 mL of dilute bacterial suspension, and incubated at 37 ^o^C for 12 h, respectively. During the incubation, the optical density (OD_600_) of the bacterial suspension was measured through a spectrophotometer at 600 nm. Finally, after the appropriate dilution of the number of wells, 0.1 mL of the bacteria suspensions were then spread on agar plates (Bizheng Biotech CO. LTD, China) containing mannitol salts and inoculated at 37 ℃. After 24 h of inoculation, the number of bacterial colonies was counted. Each measurement was performed three times.

*In vivo wound healing mode:* Full‐thickness skin defect rat model was established to assess the wound healing promoting effect of CMC-Fe/PVA@PCL-Cur gel NFSs. 6-week-old healthy male Sprague Dawley rats with an average weight of 150-230 g were obtained from Nanjing Jinling Hospital (Nanjing, China). The rats were randomly divided into 5 groups (each group contains 3 mice). First, the rats were anaesthetized by ketamine (50 mg kg^-1^ body weight) and xylazine (5 mg kg^-1^ body weight) mixture, and shaved the dorsal hair. After the routine disinfection and towel spreading, circular wounds with 1 cm of diameter were created on the back of rats, following by treated with PBS, CMC-Na/PVA@PCL nanofiber, 0.8-CMC-Fe/PVA@PCL-Cur, 1.6-CMC-Fe/PVA@PCL-Cur and 2.4-CMC-Fe/PVA@PCL-Cur gel NFSs, respectively. Then the rats were allowed to heal for 2 weeks, and the wound area was recorded during this period. Finally, the rats were executed and the granulation tissues were collected for histological and immunofluorescence analysis.

*Characterization:* The surface morphology of the gel NFSs were tested by Scanning Electron Microscopy (SEM) with a Quanta 200 (Philips-FEI, Netherlands) instrument at 5 kV. JEOL JEM-2010 TEM was used to observe the HRTEM of the gel NFSs. Atomic force microscopy (AFM) images were accomplished under ambient conditions with Bruker Dimension Icon scanning probe microscope in the tapping mode of operation. The chemical structure of gel NFSs was characterized by FT-IR spectroscopy through Nicolet 6700 spectrometer. Gel formation and drug loading processes were recorded through micro-IR images by using a Thermo Scientific Nicolet iN10 infrared microscope (Thermo Electron Corporation, USA). The water contact angle was measured with a contact angle tester (DSA100, KRÜSS, Germany). The mechanical properties of gel NFSs were tested by using a SANS CMT6203 testing machine.

*Ethical Certiﬁcation:* All animal experiments in our study were performed according to the principles of the Declaration of Helsinki, and were approved by the Institutional Animal Care and Use Committee of the Drug Safety Evaluation Center of Jiangsu Province (SYXK(SU)2021-0040).

*Statistical Analysis:* The statistical information was displayed as mean ± standard deviation (SD). One-way analysis of variance or the unpaired Student’s t-test were used to evaluate the significance between groups. At p < 0.05 (*p < 0.05, **p < 0.01, ***p < 0.001), the differences were deemed significant. The statistical analysis and visualizations were created using OriginPro 2020 and GraphPad Prism version 7. At least five fields were taken for each section during the histological evaluation, and Image J software was used to evaluate the photos.

**Supporting Figures**


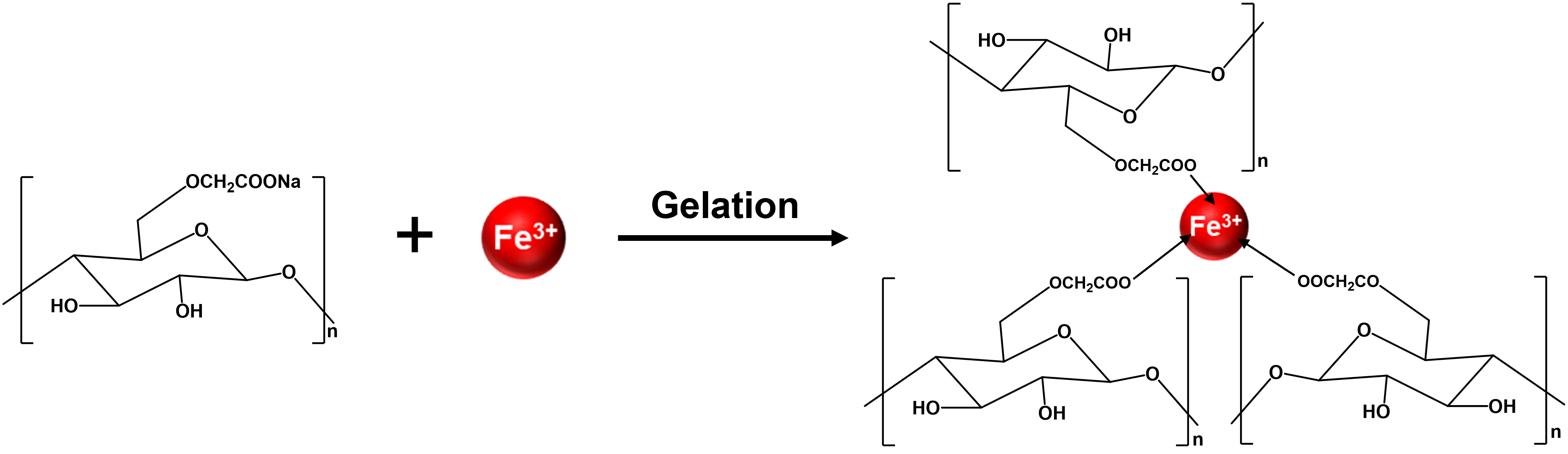


**Figure S1.** The formation mechanism of CMC-Fe gel shell.


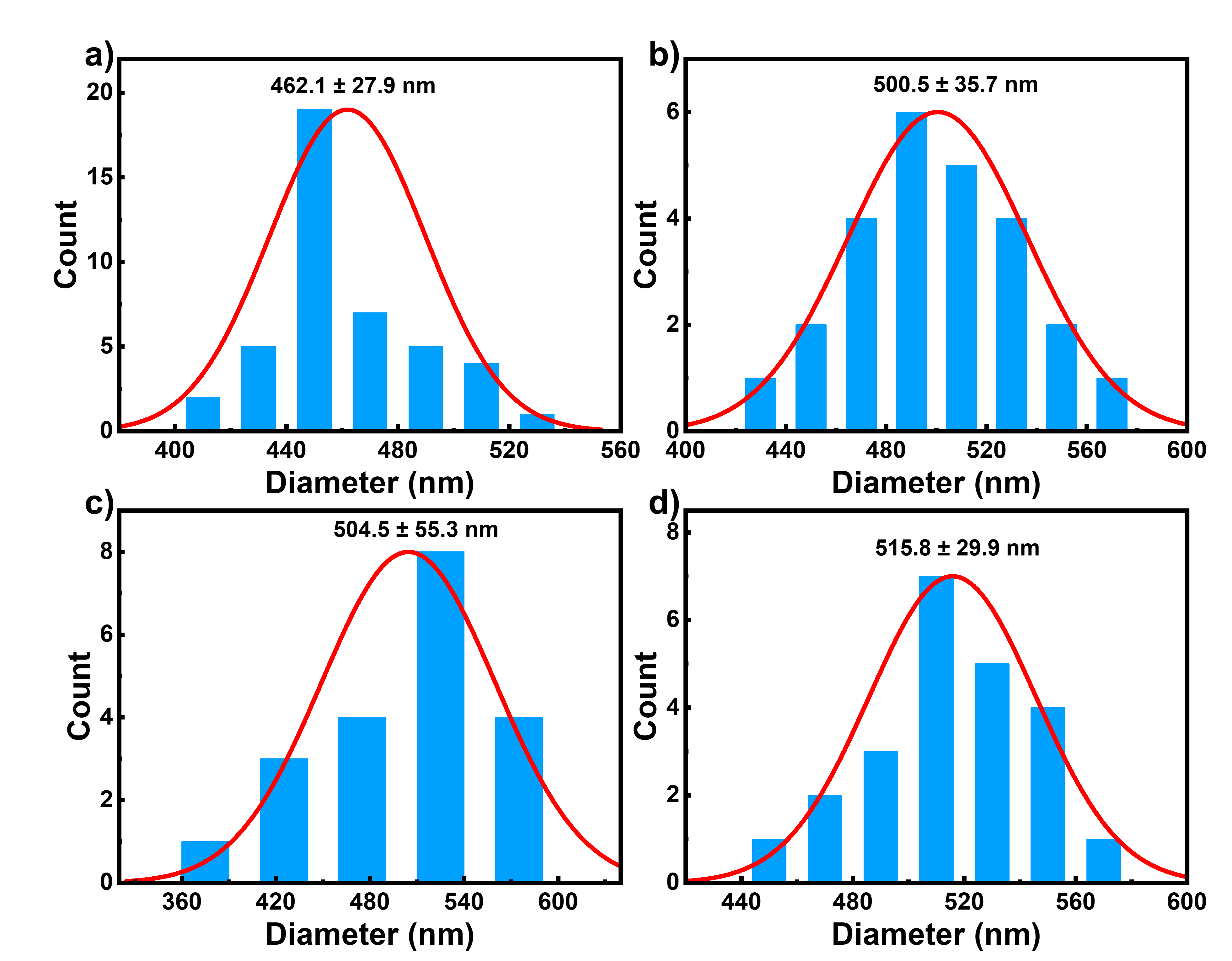


**Figure S2.** Diameter distribution of a) CMC-Na/PVA@PCL nanofiber, b) 0.8-CMC-Fe/PVA@PCL-Cur gel NFSs, c) 1.6-CMC-Fe/PVA@PCL-Cur gel NFSs and d) 2.4-CMC-Fe/PVA@PCL-Cur gel NFSs.


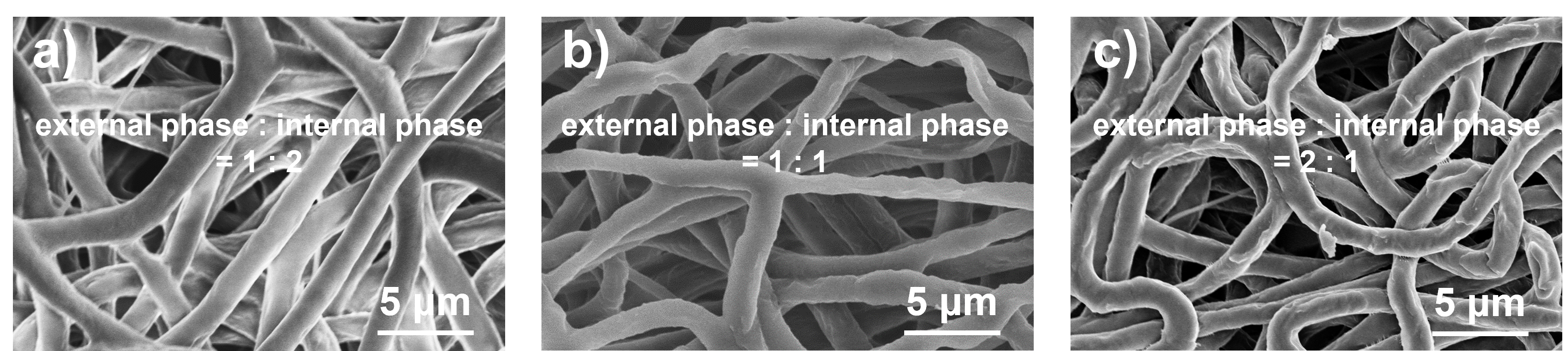


**Figure S3.** SEM images of gel NFSs prepared under different flow ratios of external and internal phases: a) 1 : 2, b) 1 : 1, c) 2 : 1.


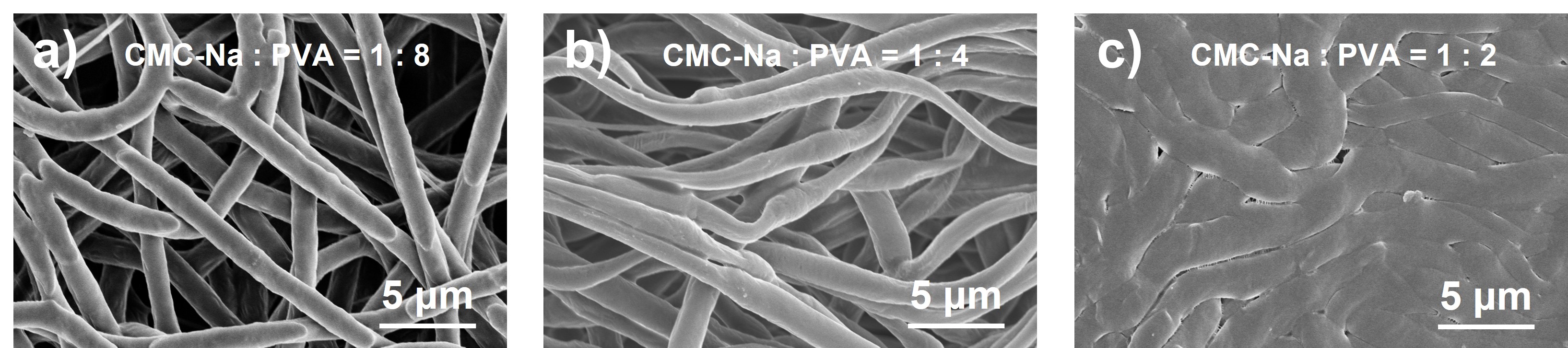


**Figure S4.** SEM images of gel NFSs prepared under different mass ratios of CMC-Na and PVA: a) 1 : 8, b) 1 : 4, c) 1 : 2.


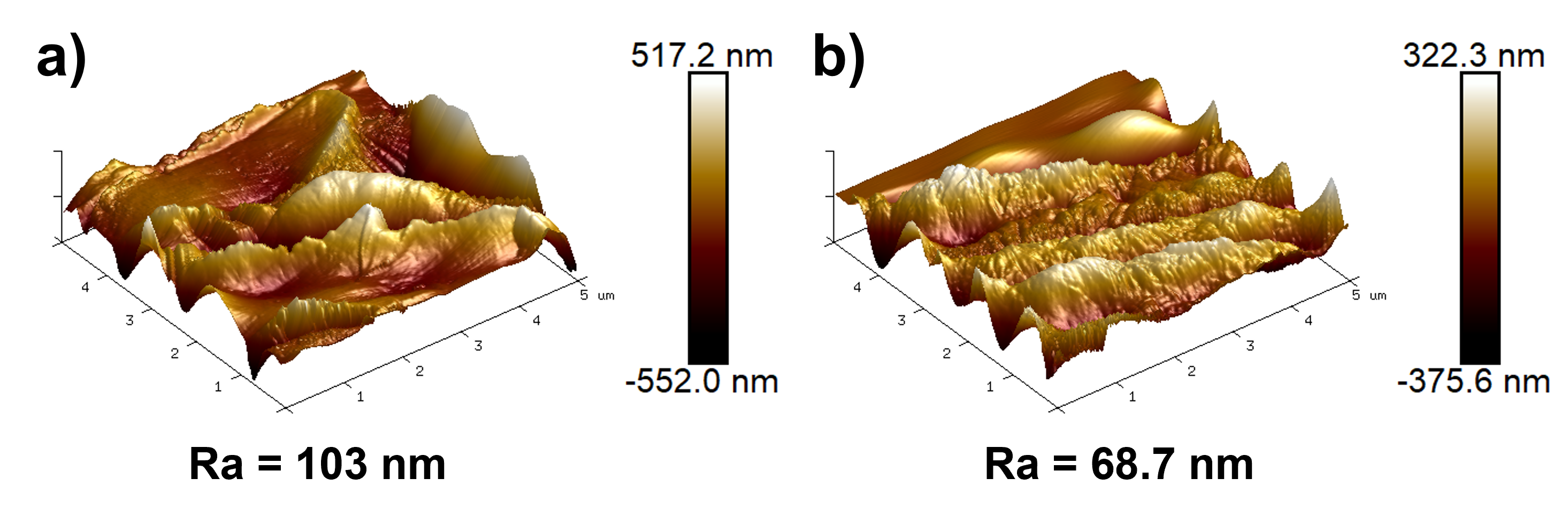


**Figure S5.** AFM images of the surface view of a) CMC-Na/PVA@PCL nanofiber and b) 2.4-CMC-Fe/PVA@PCL-Cur gel NFSs.


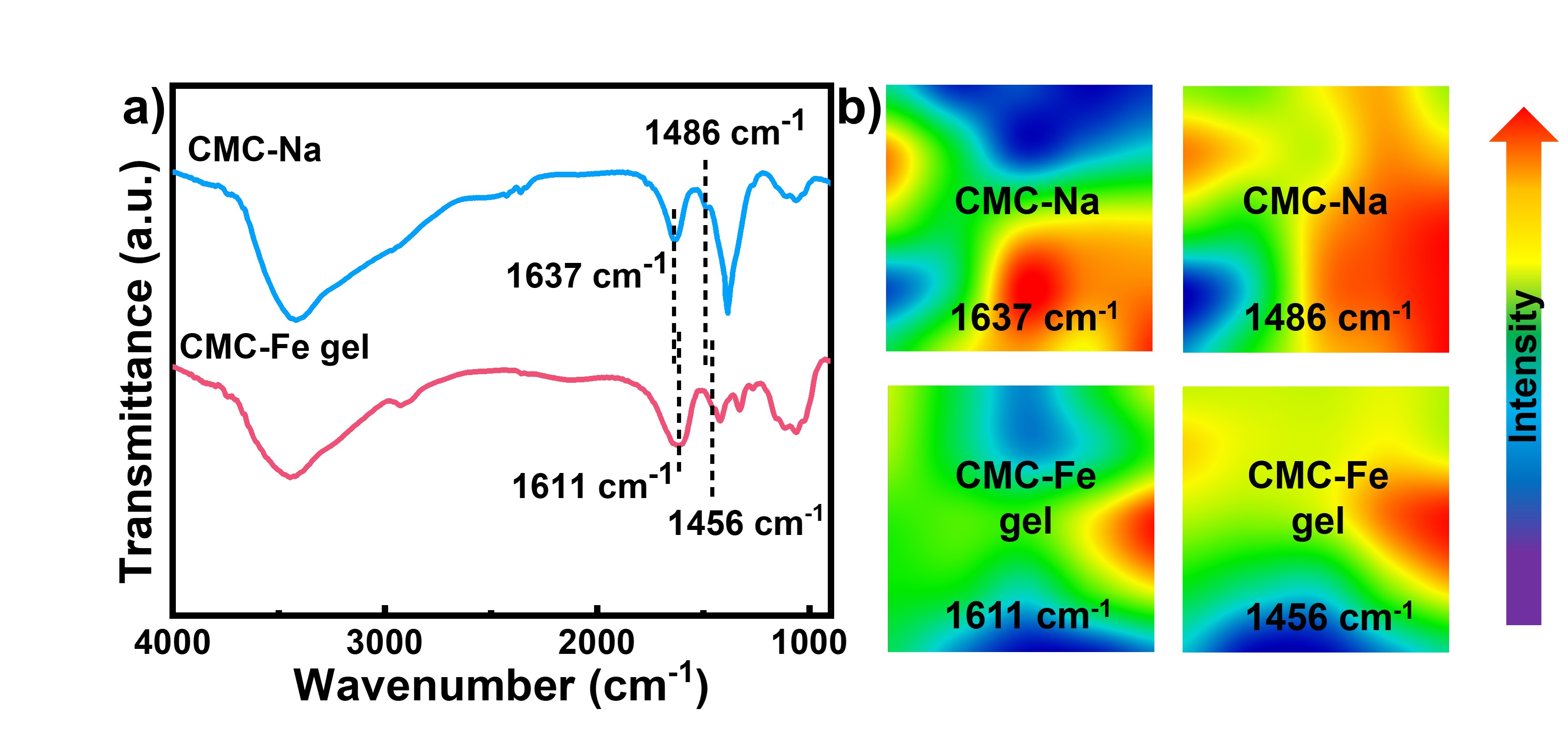


**Figure S6.** a) FT-IR spectra of CMC-Na and CMC-Fe gel and b) micro-IR images of the intensities of -COO^-^ (asymmetric stretching vibration peak and symmetric stretching vibration peak).


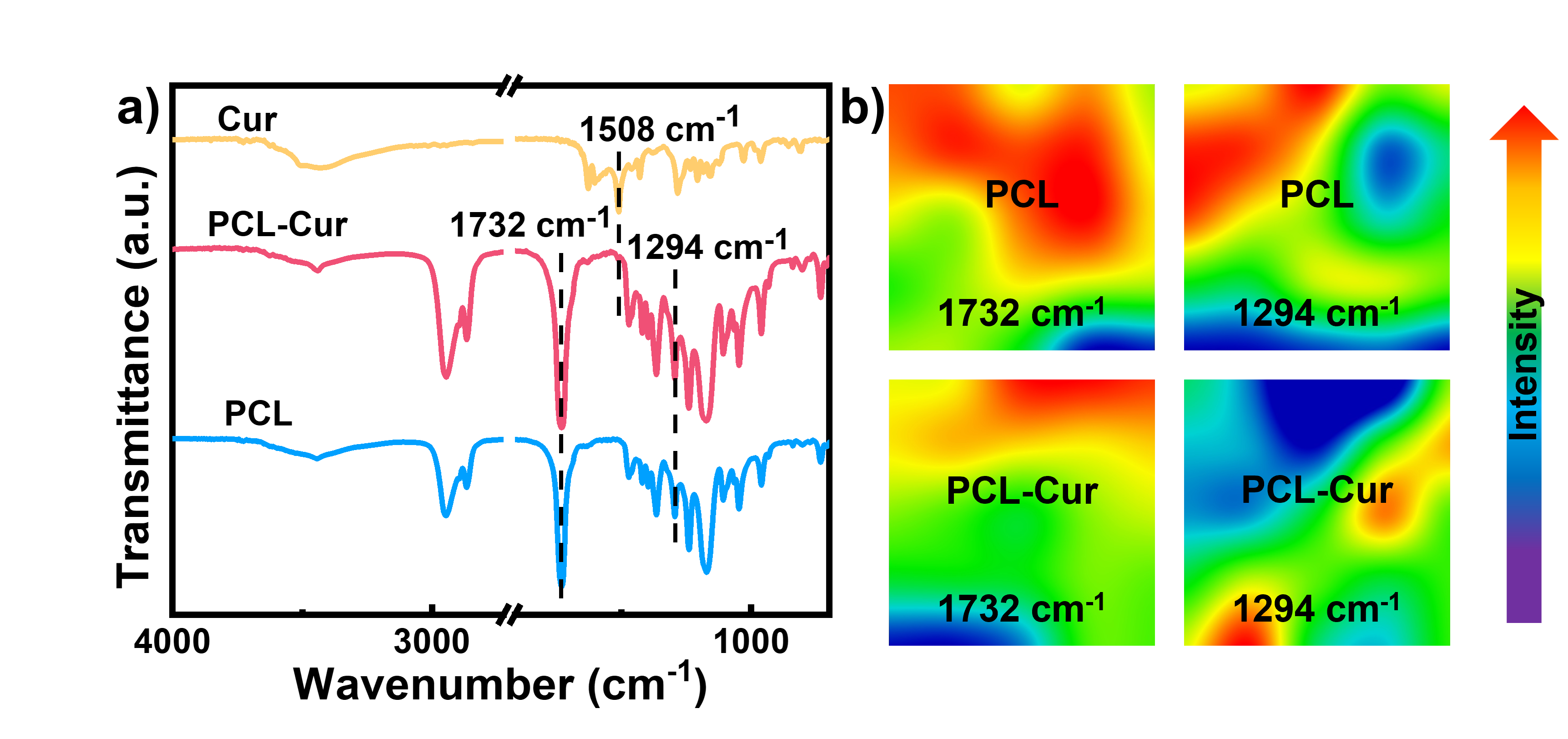


**Figure S7.** a) FT-IR spectra of pure Cur, pure PCL, PCL-Cur and b) micro-IR images of the intensities of -C=O (characteristic absorption peak at 1732 cm^-1^) and -COO^-^ (characteristic absorption peak at 1294 cm^-1^) of PCL and PCL-Cur.


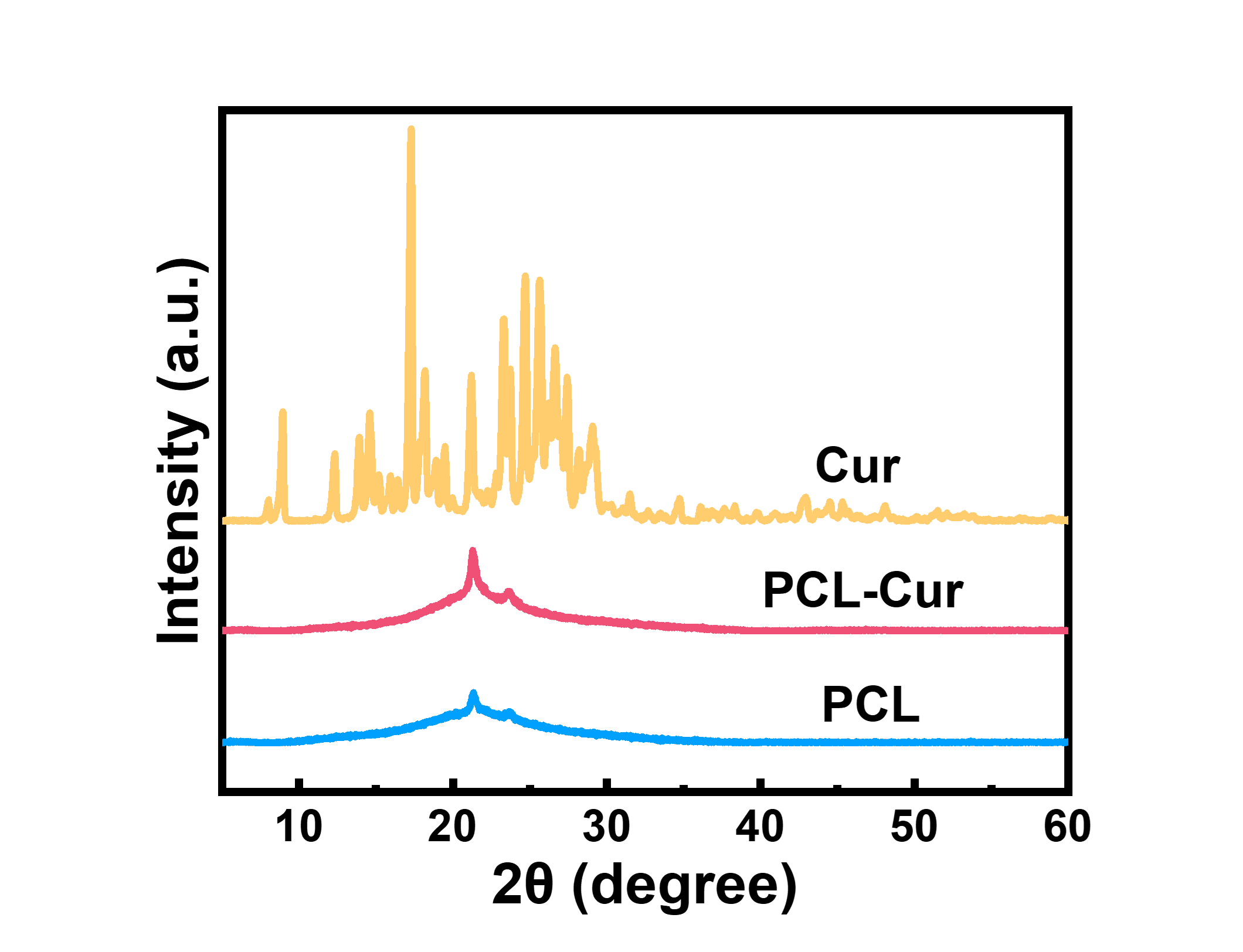


**Figure S8.** XRD spectra of pure Cur, pure PCL and PCL-Cur.


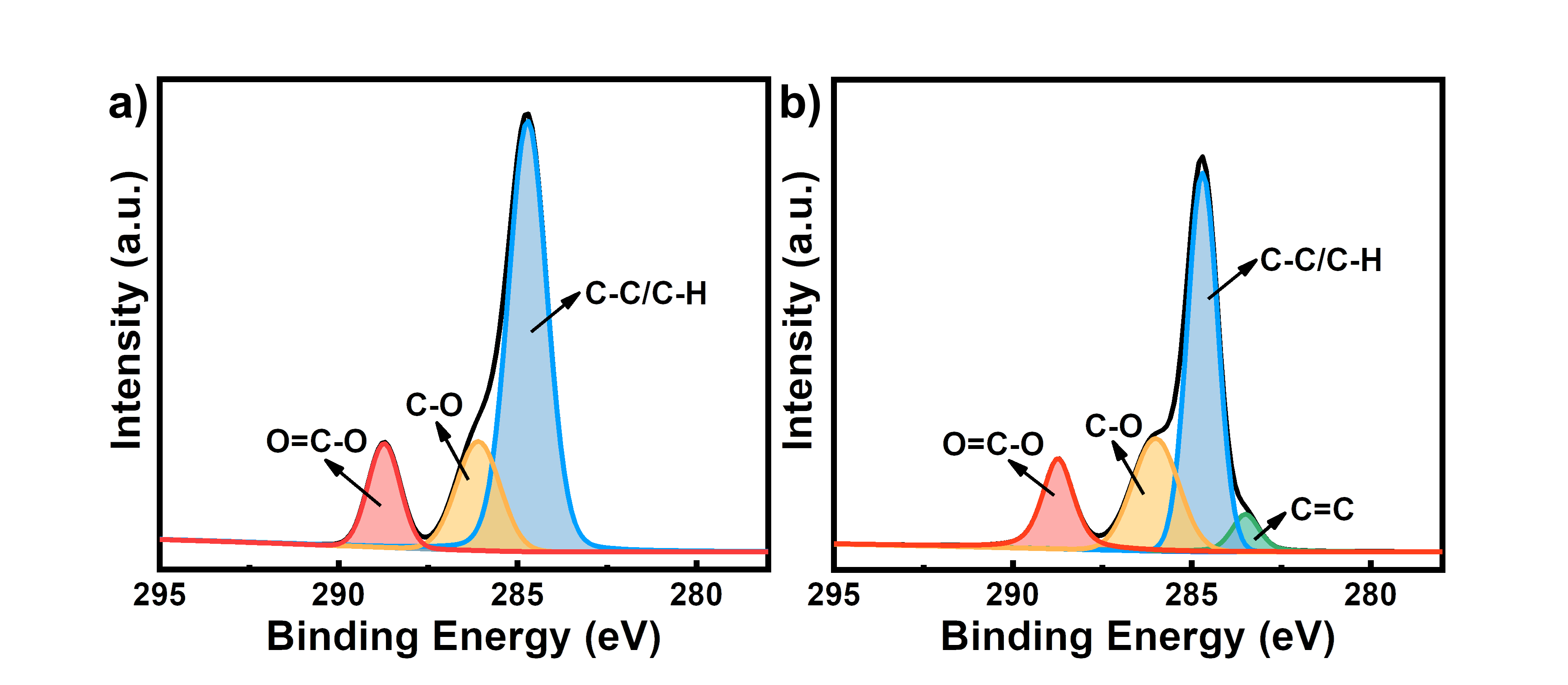


**Figure S9.** C 1s XPS spectra of a) PCL and b) PCL-Cur.


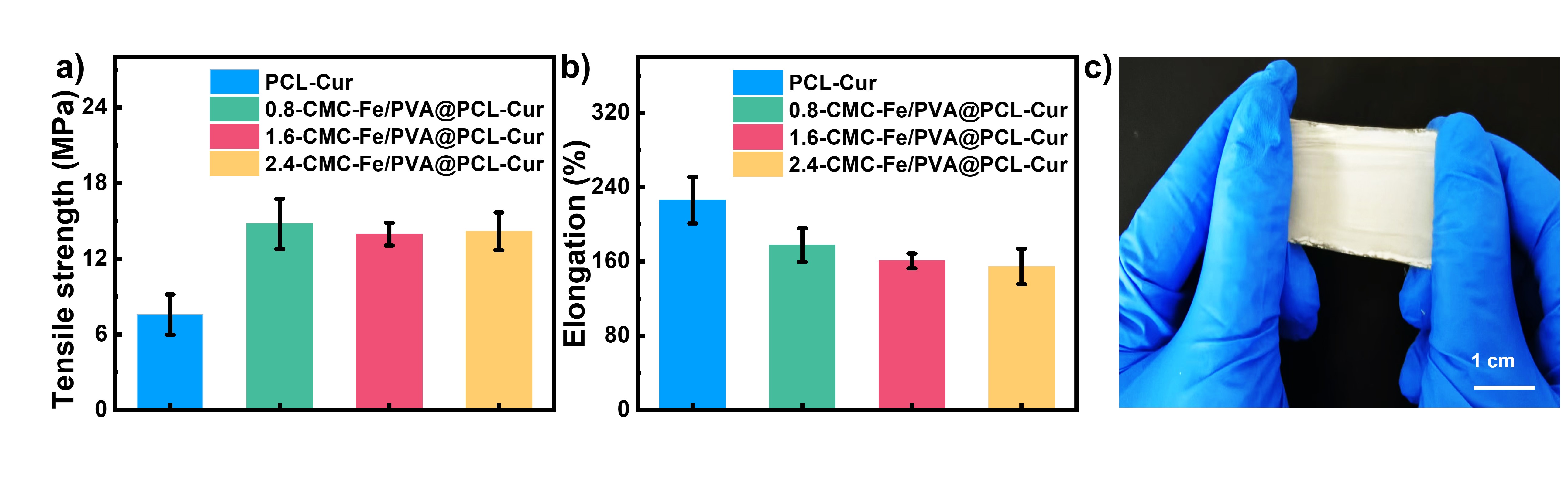


**Figure S10.** a) Tensile strength of pure PCL-Cur nanofiber and gel NFSs. b) Elongation of PCL-Cur nanofiber and gel NFSs. c) Photograph of the gel NFSs.


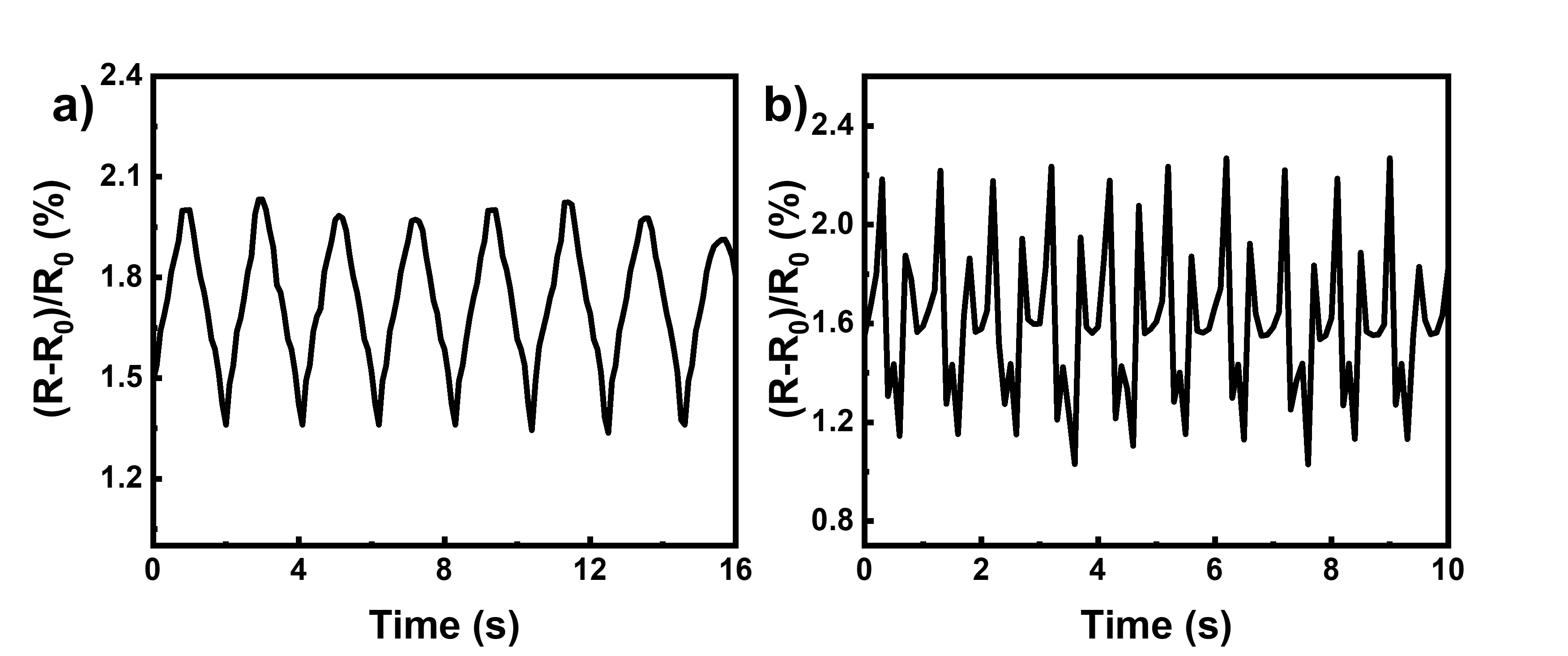


**Figure S11.** Electrical signal profile of gel NFSs in monitoring a) knee bending and b) cheek vibration.


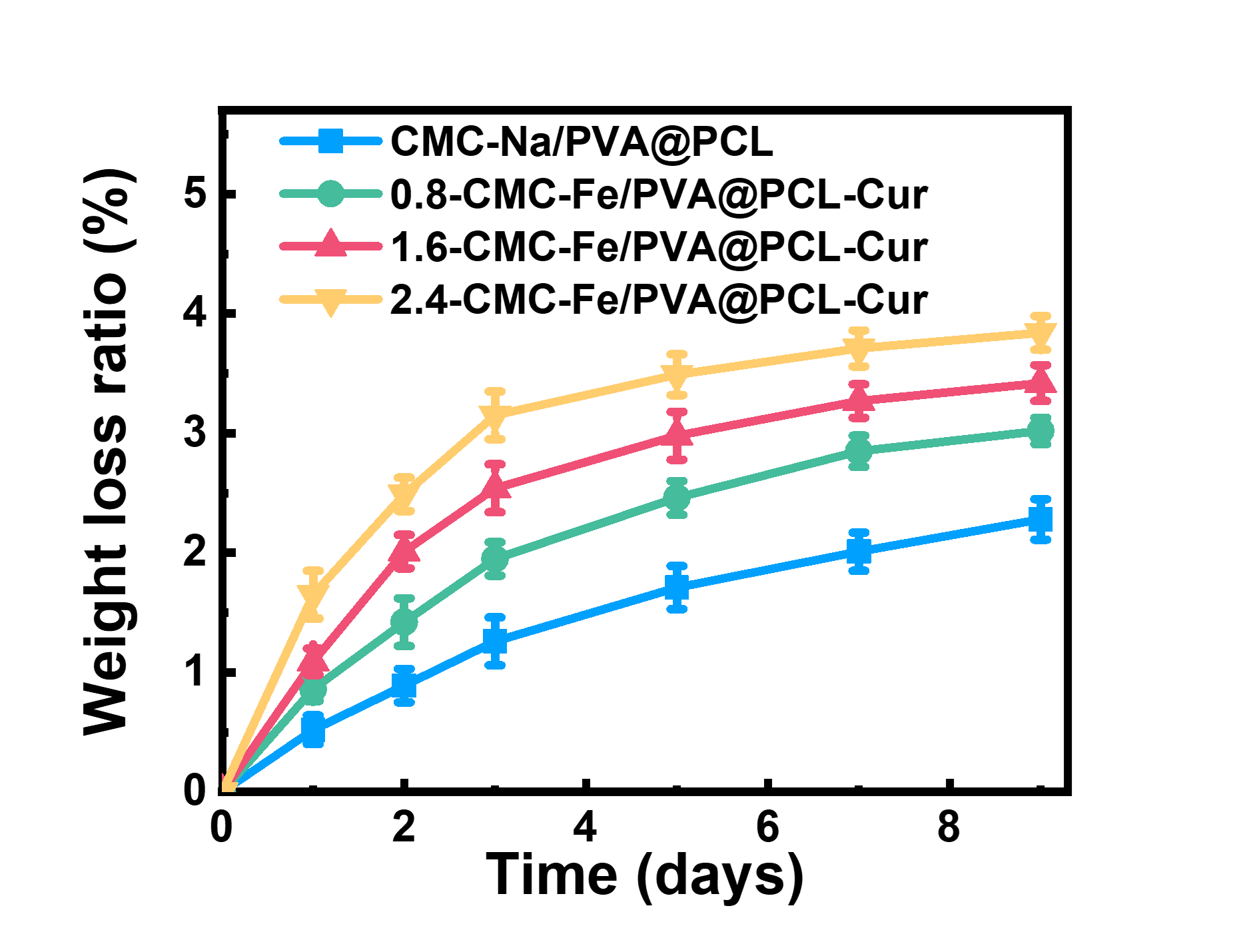


**Figure S12.** Weight loss ratio of CMC-Na/PVA@PCL nanofiber and gel NFSs.


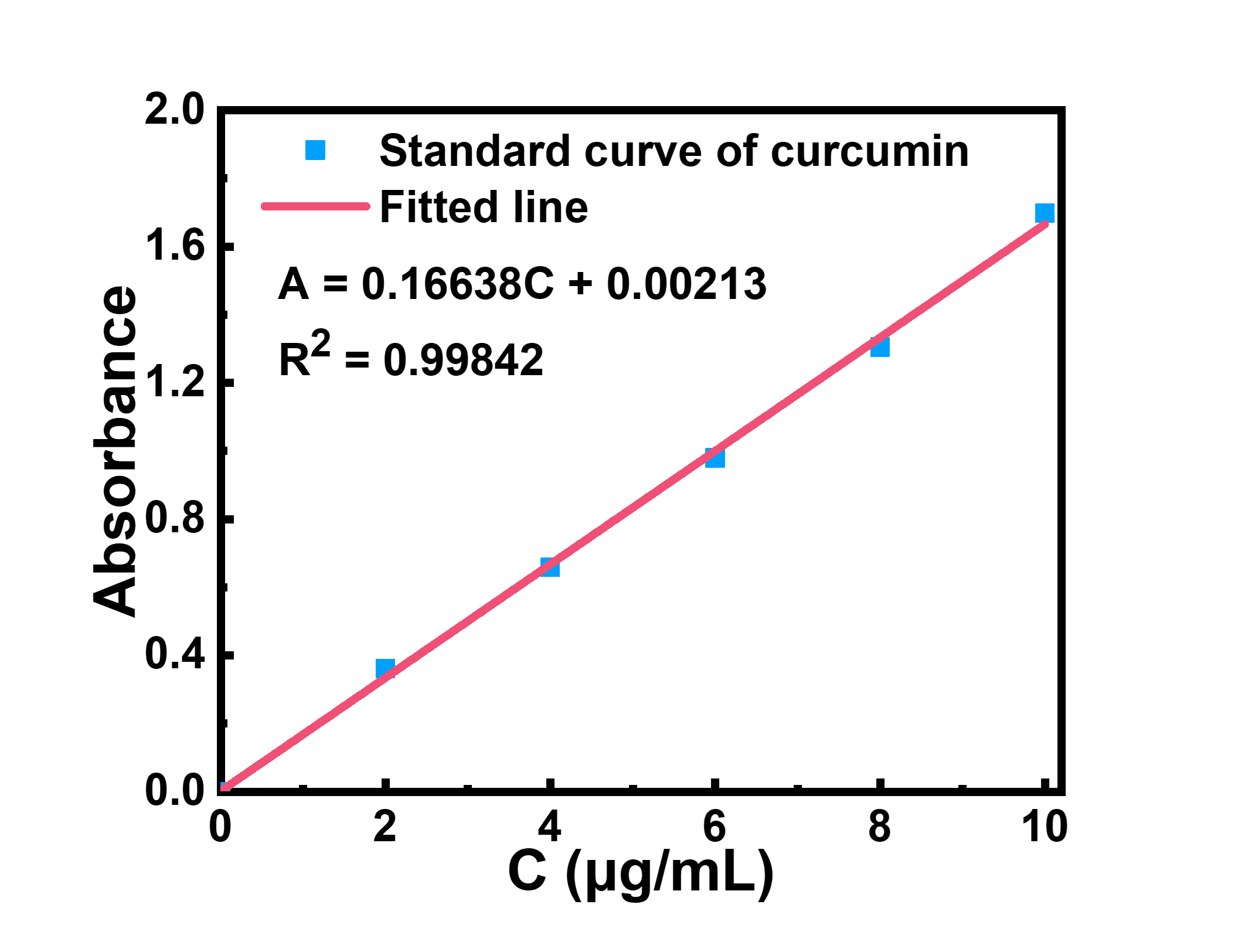


**Figure S13.** Standard curve for Cur release.

To investigate the law of drug release in vitro of gel NFSs, a standard curve of Cur concentration versus the UV absorbance was obtained as followed equation:

| $A=0.16638C+0.00213 (R^{2}=0.99842)$ | (4) |
| --- | --- |

where *A* represents the UV absorbance and *C* represents the concentration of Cur in the PBS solution.


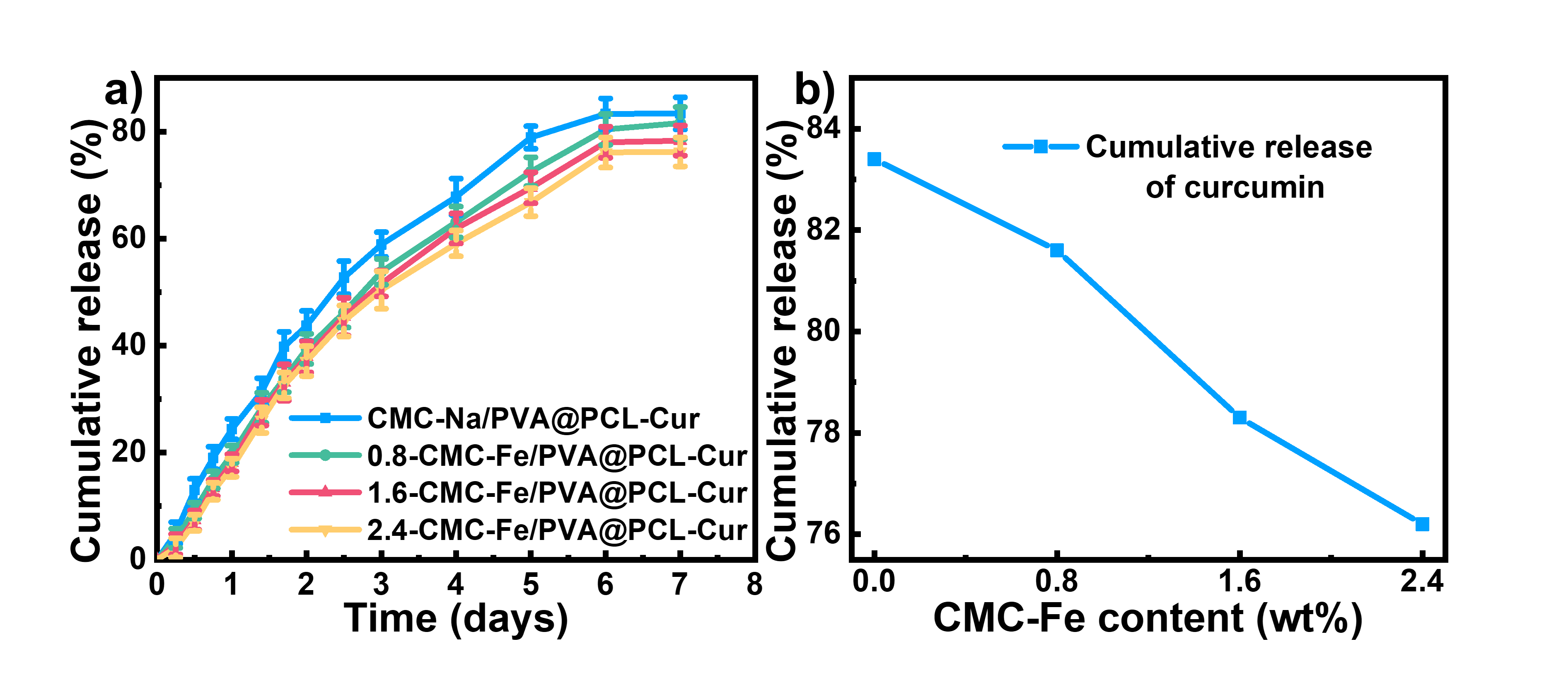


**Figure S14.** a) Cur release curve of gel NFSs in PBS solution. b) Cumulative release of Cur from gel NFSs with different CMC-Fe content within 7 days.


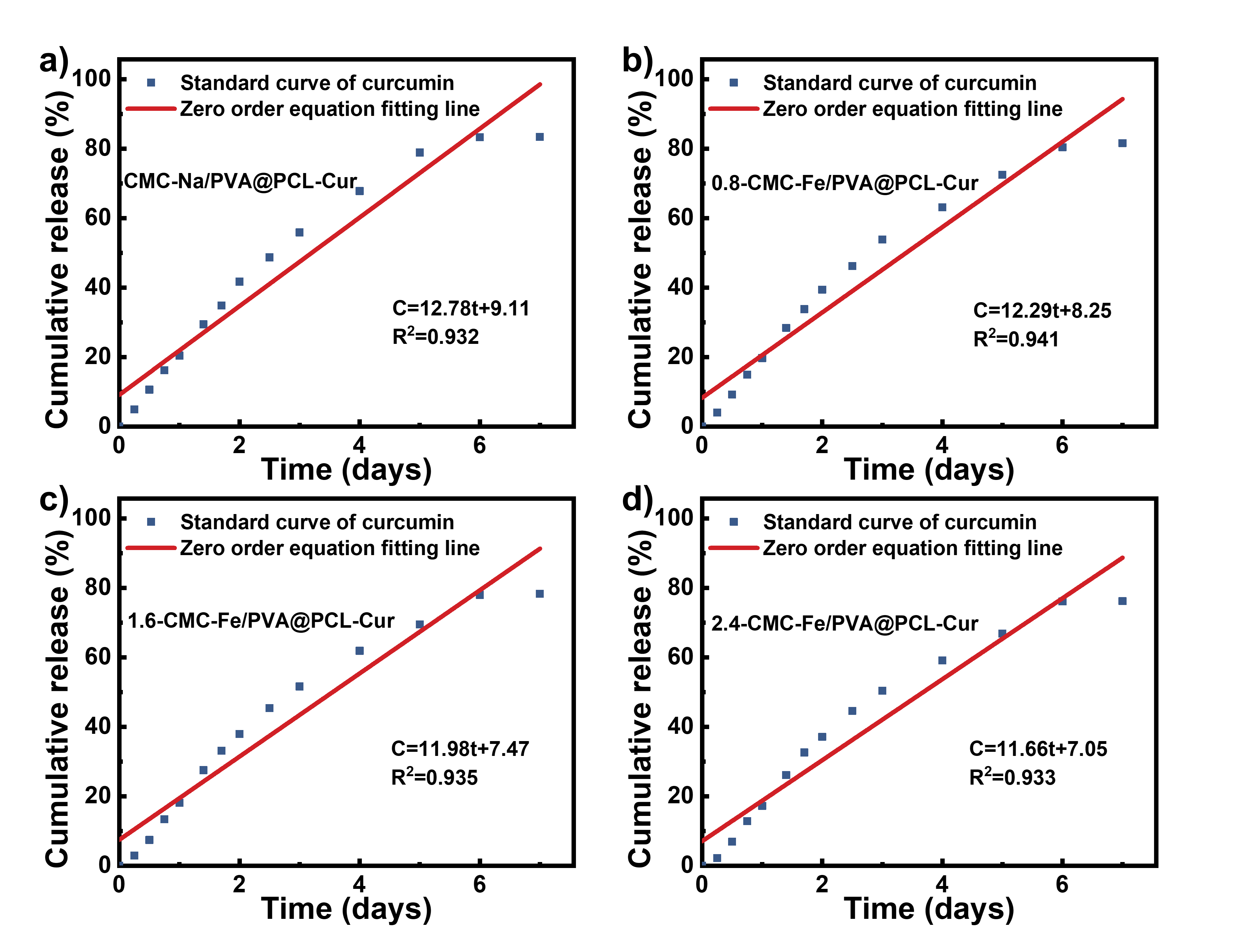


**Figure S15.** Drug release kinetics equation (Zero order equation) fitting for a) CMC-Na/PVA@PCL-Cur nanofiber, b) 0.8-CMC-Fe/PVA@PCL-Cur gel NFSs, c) 1.6-CMC-Fe/PVA@PCL-Cur gel NFSs and d) 2.4-CMC-Fe/PVA@PCL-Cur gel NFSs. (*C* represents the cumulative release of Cur, *R^2^* represents the correlation coefficients)


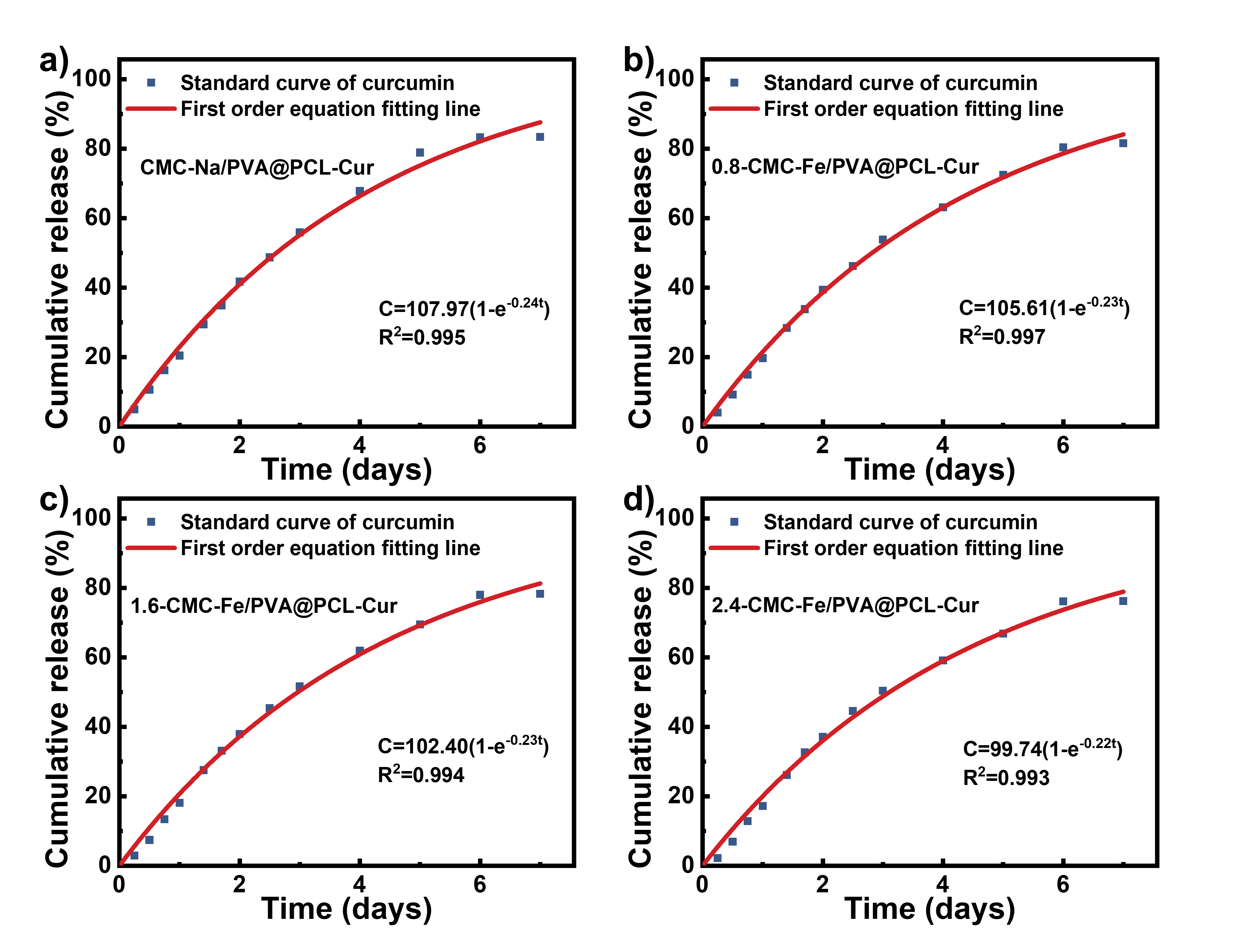


**Figure S16.** Drug release kinetics equation (First order equation) fitting for a) CMC-Na/PVA@PCL-Cur nanofiber, b) 0.8-CMC-Fe/PVA@PCL-Cur gel NFSs, c) 1.6-CMC-Fe/PVA@PCL-Cur gel NFSs and d) 2.4-CMC-Fe/PVA@PCL-Cur gel NFSs. (*C* represents the cumulative release of Cur, *R^2^* represents the correlation coefficients)


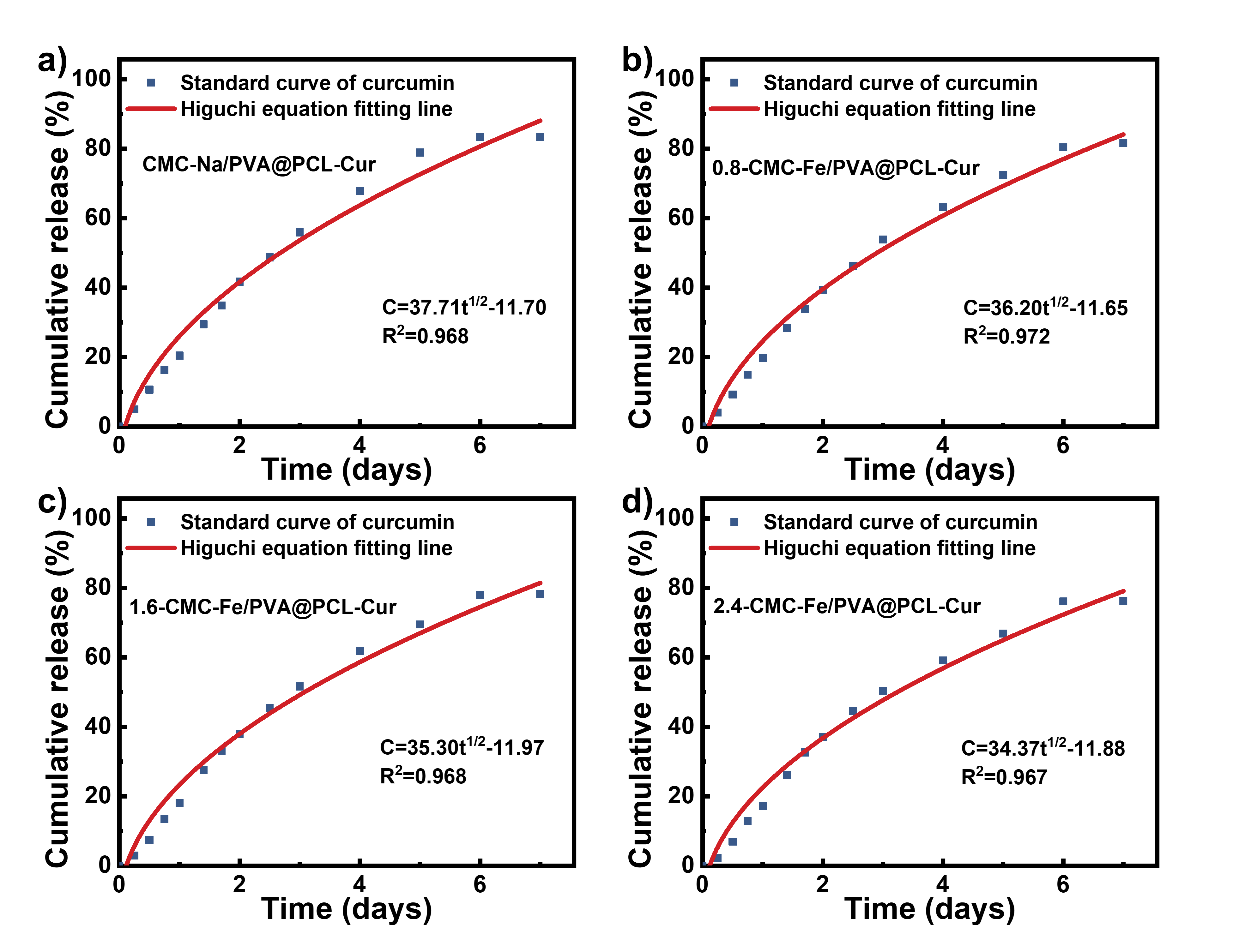


**Figure S17.** Drug release kinetics equation (Higuchi equation) fitting for a) CMC-Na/PVA@PCL-Cur nanofiber, b) 0.8-CMC-Fe/PVA@PCL-Cur gel NFSs, c) 1.6-CMC-Fe/PVA@PCL-Cur gel NFSs and d) 2.4-CMC-Fe/PVA@PCL-Cur gel NFSs. (*C* represents the cumulative release of Cur, *R^2^* represents the correlation coefficients)

**Table S1.** Correlation coefficients of mathematical dynamical models for Cur release in CMC-Na/PVA@PCL-Cur nanofiber and CMC-Fe/PVA@PCL-Cur gel NFSs.

| Samples | Zero-order equation (R^2^) | First-order equation (R^2^) | Higuchi equation (R^2^) |
| --- | --- | --- | --- |
| CMC-Na/PVA  @PCL-Cur | 0.932 | 0.995 | 0.968 |
| 0.8-CMC-Fe/PVA  @PCL-Cur | 0.941 | 0.997 | 0.972 |
| 1.6-CMC-Fe/PVA  @PCL-Cur | 0.935 | 0.994 | 0.968 |
| 2.4-CMC-Fe/PVA  @PCL-Cur | 0.933 | 0.993 | 0.967 |


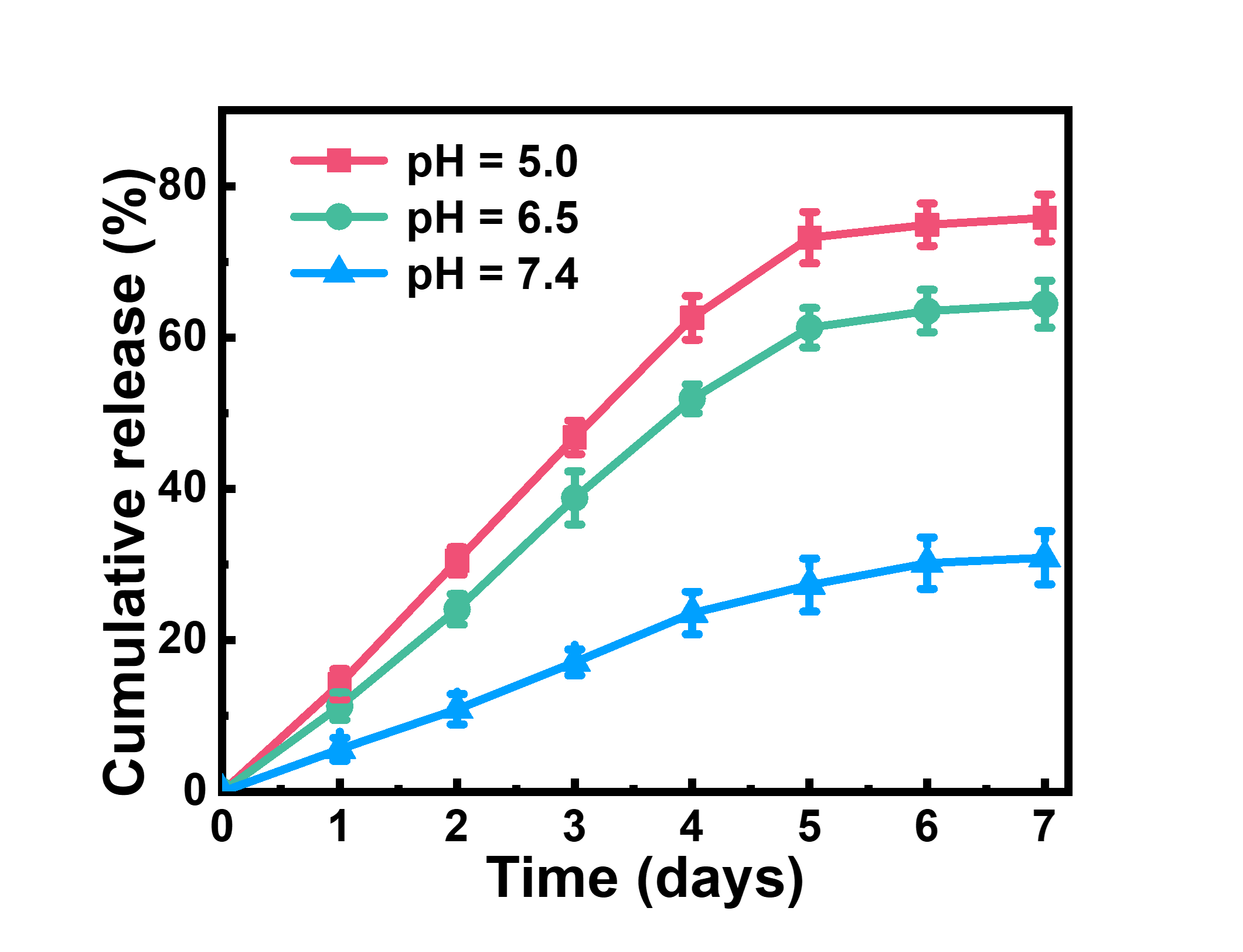


**Figure S18.** Drug release performance of 2.4-CMC-Fe/PVA@PCL-Cur gel NFSs at different pH values.


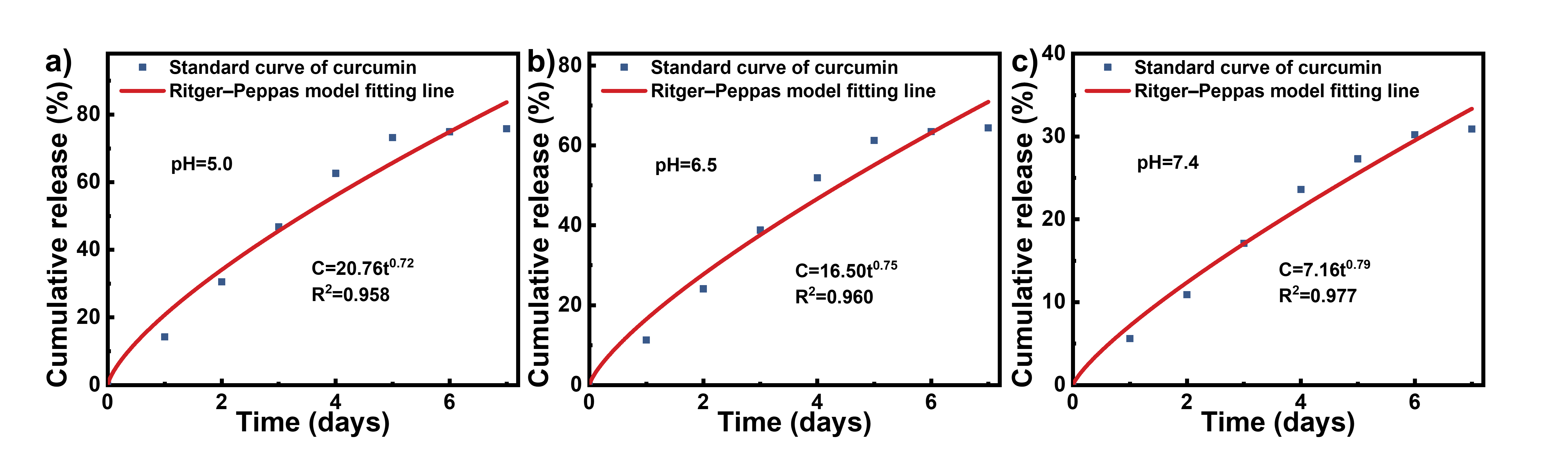


**Figure S19.** Kinetic fitting of drug release from 2.4-CMC-Fe/PVA@PCL-Cur gel NFSs at a) pH = 5.0, b) pH = 6.5 and c) pH = 7.4, respectively.

**Table S2.** Kinetic equation fitting of Cur release from 2.4-CMC-Fe/PVA@PCL-Cur gel NFSs at different pH values.

| Samples | pH | k | n | R^2^ |
| --- | --- | --- | --- | --- |
| 2.4-CMC-Fe/PVA  @PCL-Cur | 5.0 | 20.76 | 0.72 | 0.958 |
| 2.4-CMC-Fe/PVA  @PCL-Cur | 6.5 | 16.50 | 0.75 | 0.960 |
| 2.4-CMC-Fe/PVA  @PCL-Cur | 7.4 | 7.16 | 0.79 | 0.977 |
